# Selection and Validation of Reference Genes for Normalisation of Gene Expression in *Glehnia littoralis*

**DOI:** 10.1038/s41598-020-63917-5

**Published:** 2020-04-30

**Authors:** Li Li, Naiwei Li, Hailing Fang, Xiwu Qi, Yifeng Zhou

**Affiliations:** 1 Jiangsu Key Laboratory for the Research and Utilization of Plant Resources, Institute of Botany, Jiangsu Province and Chinese Academy of Sciences (Nanjing Botanical Garden Mem. Sun Yat-Sen), Nanjing, Jiangsu China; 2 The Jiangsu Provincial Platform for Conservation and Utilization of Agricultural Germplasm, Nanjing, Jiangsu China

**Keywords:** Plant molecular biology, Biotechnology

## Abstract

*Glehnia littoralis* is an important medicinal halophyte—the dried root of which is used as Chinese herbal medicine. However, the use, selection and stability of reference genes are rarely verified in studies of *G. littoralis*, which hampers investigation of its salt tolerance and metabolism. In this study, we selected 13 candidate reference genes from the transcriptome data of *G. littoralis*—serine/threonine-protein phosphatase PP2A (*PP2A*), polyubiquitin 10 (*UBQ10*), actin (*ACT*), elongation factor 1-α (*EF1-α*), glyceraldehyde-3-phosphate dehydrogenase (*GAPDH*), α-tubulin (*α-TUB*), β-tubulin (*β-TUB*), polypyrimidine tract-binding protein 1 (*PTBP1*), expressed protein 1 (*EXP1*), expressed protein 2 (*EXP2*), TIP41-like (*TIP41*), SAND family (*SAND*), and cyclophilin 2 (*CYP2*), and used qRT-PCR to analyse their expression levels in roots of *G. littoralis* treated with NaCl, polyethylene glycol (PEG), abscisic acid (ABA), and methyl jasmonate (MeJA), as well as in various organs of *G. littoralis*. The ΔCt, geNorm, NormFinder, and BestKeeper algorithms were used to assess the expression stability of the candidate reference genes and the results were then used to generate a comprehensive rank list with the RankAggreg R package. The most stable reference genes for normalisation were *EXP1* and *PP2A* in response to NaCl, *EXP2* and *PP2A* in response to ABA, *CYP2* and *α-TUB* in response to MeJA, and *ACT* and *EXP1* in the PEG and the organ subsets. *GAPDH*, *β-TUB*, and *UBQ10* exhibited low stability and so were unsuitable for normalisation. This study is the first systematic analysis of candidate reference genes in *G. littoralis* and will facilitate further investigation of normalisation of gene expression in *G. littoralis*.

## Introduction

*Glehnia littoralis* Fr. Schmidt ex Miq. is an herbaceous perennial in Family Umbelliferae. It is a medicinal halophyte naturally distributed in coastal areas, such as eastern China, Japan, the Korean Peninsula, Russia, and the United States^1^. Peeled and dried *G. littoralis* roots, named Radix Glehniae, are commonly used as a traditional Chinese herbal medicine for moistening the lungs and removing lung-heat; relieving thirst, dry throat and cough; and curing gastrointestinal disorders^2,3^. Radix Glehniae is rich in coumarins, coumarin glycosides, phospholipids, and polysaccharides, and is a component of anti-aging, anti-inflammation, and health-promoting agents^2–4^. The annual demand for Radix Glehniae is more than 8,000 tons. Due to the destruction of the ecological environment of beaches, *G. littoralis*-producing regions have shrunk or disappeared and *G. littoralis* cultivation has moved inland, resulting in an insufficient supply of uneven quality. Therefore, coastal cultivation of *G. littoralis* must be restored to guarantee the quality of medicinal materials. However, due to long-term inland cultivation and artificial selection, *G. littoralis* has insufficient salt tolerance and the mechanisms underlying its salt tolerance are unclear. Investigation of the salt-tolerance mechanism of *G. littoralis* would assist in restoring coastal production. High salinity affects the accumulation of secondary metabolites in plants^5–7^; for example, the contents of polyphenols and carotenoids in buckwheat, flavonoids in barley, and polyphenols in *Cakile maritima* increased after salt treatment^8–10^. The coastal salt environment of *G. littoralis* likely impacts the accumulation of secondary metabolites; indeed, accumulation of furocoumarins was increased by salt stress in *G. littoralis*^11^. However, the underlying mechanism is unknown. Studies of the salt tolerance of *G. littoralis* and its effect on the synthesis and accumulation of secondary metabolites are needed to promote production of high-quality medicinal materials, improve the quality of germplasm resources, and facilitate investigation of the function and regulation of genes in plants.

Quantitative real-time PCR (qRT-PCR) is used to analyse the expression patterns of plant genes because of its high sensitivity, quantitative accuracy, low cost, and high throughput^12–14^. To eliminate the influence of different templates and reverse-transcription efficiencies, stable reference genes are used to normalise gene expression values determined by qRT-PCR. However, the expression levels of reference genes differ among environmental conditions, tissues, organs, and developmental stages. They can only remain relatively stable within a range of experimental conditions. The use of a reference gene irrespective of the experimental conditions will affect the reliability of the results. Housekeeping genes such as *18S rRNA*, glyceraldehyde-3-phosphate dehydrogenase (*GAPDH*), actin (*ACT*), and tubulin (*TUB*) are commonly used as reference genes in plants^15–17^. Novel reference genes, such as eukaryotic initiation factor 4α (*eIF-4α*), serine/threonine-protein phosphatase PP2A (*PP2A*), SAND family (*SAND*), and TIP41-like (*TIP41*), have also been used for normalisation^18–20^. However, no reference gene of *G. littoralis* has been assessed under normal and abnormal conditions, which hampers research into its metabolic pathways and mechanism of salt tolerance.

We previously performed a comprehensive transcriptome analysis of *G. littoralis* in response to salt stress via RNA sequencing (RNA-seq)^2^. The RNA-seq results identified a large number of unigenes involved in the response to salt stress in *G. littoralis*. In the present study, we selected 13 candidate reference genes based on the transcriptome data of *G. littoralis* and evaluated their expression levels using qRT-PCR in response to salt (NaCl), drought (PEG), abscisic acid (ABA), and methyl jasmonate (MeJA), and in various organs. We also evaluated the expression stability of the candidate reference genes using the statistical algorithms ΔCt^21^, geNorm^22^, NormFinder^23^, and BestKeeper^24^, and performed a comprehensive stability ranking^25^. Finally, we validated the candidate reference genes using several stress-related genes from *G. littoralis* transcriptome data. This work provides the first systematic analysis of candidate reference genes in *G*. *littoralis*, and the results will facilitate future studies on gene expression in *G*. *littoralis*, as well as in other species of Umbelliferae.

## Results

### Selection of candidate reference genes, primer specificity, and amplification efficiency

Based on the transcriptome data of *G*. *littoralis* obtained in our previous study^2^, thirteen candidate reference genes were selected with reference to the reference genes of *Arabidopsis thaliana*, which are frequently used in studies based on qRT-PCR^18^. The candidate reference genes were *PP2A*, polyubiquitin 10 (*UBQ10*), *ACT*, elongation factor 1-α (*EF1-α*), *GAPDH*, *α-TUB*, *β-TUB*, polypyrimidine tract-binding protein 1 (*PTBP1*), expressed protein 1 (*EXP1*), expressed protein 2 (*EXP2*), *TIP41*, *SAND*, and cyclophilin 2 (*CYP2*). The sequences of the 13 candidate reference genes from RNA-seq are listed in Supplementary File S1 and their characteristics and qRT-PCR primer sequences are shown in Table 1. Primer specificity was evaluated via agarose gel electrophoresis, sequencing, and melting curve analysis. A single DNA band at the correct molecular weight and a single peak in the melting curve for each gene indicated high primer specificity (Supplementary Figs. S1 and S2). The electropherograms of sanger sequencing for qRT-PCR amplicons obtained from 13 reference genes are listed in Supplementary File S2. The amplification efficiency (*E*) and correlation coefficient (R^2^) of each candidate reference gene were calculated from calibration curves with R^2^ values of > 0.99; values of *E* ranged from 88% to 108% (Table 1).

**Table 1:** Genes and primer sets of the candidate reference genes used for qRT-PCR.

Gene name	Gene symbol	Gene ID	Arabidopsis homolog locus	Primer sequence (forward/reverse)	Size (bp)	E(%)	R^2^
Serine/threonine-protein phosphatase PP2A	*PP2A*	comp29669_c0_seq3	AT1G13320	GCAACCATTGAACCTGCTCA	199	94.17	0.9979
				GAACACGCCACGACTTATCC			
Polyubiquitin 10	*UBQ10*	comp35437_c0_seq1	AT4G05320	TGAGGGGTGGAATGCAGATT	182	94.99	0.9964
				TGCAAGTGTACGACCATCCT			
Actin	*ACT*	comp33336_c0_seq7	AT3G18780	ACCATCACCAGAATCCAGCA	195	90.36	0.9961
				CTTCGAGTTGCTCCTGAGGA			
Elongation factor 1-α	*EF1-α*	comp33645_c0_seq1	AT1G07920	AAGGATGGGCAAACTCGTGA	183	88.19	0.9994
				AGCAATTTTGTCGGGGTTGT			
Glyceraldehyde-3-phosphate dehydrogenase	*GAPDH*	comp34780_c0_seq4	AT1G13440	ACCTTCTTTGCACCTCCCTT	134	92.95	0.9976
				GCTGTCTTTGGTTGCAGGAA			
α-tubulin	*α-TUB*	comp10019_c0_seq1	AT1G50010	ACAACTTTGCTCGTGGACAC	132	91.17	0.9979
				TGCCTCCTCCAACAGCATTA			
β-tubulin	*β-TUB*	comp29155_c0_seq2	AT5G12250	CAGGTACACTCAATGCACGG	226	93.44	0.997
				CGCACCTTGAAGCTTACCAC			
Polypyrimidine tract-binding protein 1	*PTBP1*	comp32255_c0_seq25	AT3G01150	GGCTACGGTGTGATTGGAAC	226	107.22	0.9996
				TCGGCTTTTGGATTTGTGCA			
Expressed protein 1	*EXP1*	comp33961_c0_seq2	AT2G32170	AGCCCTCCTGCATGTTTAGT	216	102.12	0.9978
				ACGAAGCTGGTCACTGTCAG			
Expressed protein 2	*EXP2*	comp12579_c0_seq1	AT4G33380	ACACCCATATATTGCAGCGC	223	96.26	0.9975
				CATGATGACAAGCCTGAGGG			
TIP41-like	*TIP41*	comp13746_c0_seq1	AT4G34270	AGAGTGATGCCAAGCTGTTG	149	93.79	0.9977
				GCCTCTCTCCAGCAACATTC			
SAND family	*SAND*	comp27786_c0_seq1	AT2G28390	GGACCATTTCGATACGGATCC	234	93.91	0.9952
				AAGCGTCTCTTCATCCCGAT			
Cyclophilin 2	*CYP2*	comp30894_c0_seq1	AT4G33060	GCCACTATCATTTGAGGAGACC	222	95.03	0.9993
				CGTTGTGATTGGCTTGATCG			

### Expression profiles of the candidate reference genes

**Figure 1:**
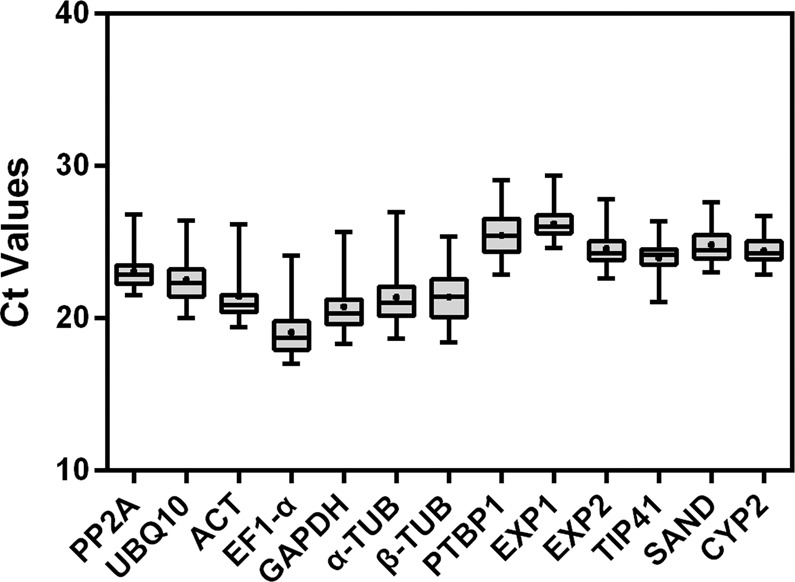
Threshold cycle (Ct) values of the candidate reference genes. Boxes indicate the 25^th^ and 75^th^ percentiles, horizontal lines represent the medians, spots indicate the means, and whiskers represent the maximum and minimum values.

We performed qRT-PCR to evaluate the expression of the 13 candidate reference genes. The expression of the candidate reference genes was determined by calculating the threshold cycle (Ct) values; the higher the Ct value, the lower the expression level. The Ct values of the 13 genes ranged from 17.02 to 29.34, with the majority ranging from 19 to 27 (Fig. 1; Supplementary Dataset 1). *EF-1a* had the lowest mean Ct value of 19.06 ± 1.47 (standard deviation [SD]), indicating that it was the most highly expressed gene, followed by *GAPDH* (20.74 ± 1.67), *a-TUB* (21.35 ± 1.58), *β-TUB* (21.4 ± 1.52), and *ACT* (21.42 ± 1.66). *UBQ10* (22.52 ± 1.34), *PP2A* (23.07 ± 1.18), *TIP41* (23.93 ± 1.05), *CYP2* (24.42 ± 0.86), *EXP2* (24.56 ± 1.15), and *SAND* (24.8 ± 1.25) were moderately expressed, and *EXP1* (26.19 ± 0.93) and *PTBP1* (25.41 ± 1.32) were expressed at low levels. *CYP2* showed the least variation in expression, and *GAPDH* and *ACT* showed the greatest variation (Fig. 1).

### Analysis of the expression stability of the candidate reference genes

To analyse the stability of the candidate reference genes, the statistical algorithms ΔCt^21^, geNorm^22^, NormFinder^23^, and BestKeeper^24^ were used.

The ΔCt method involves comparing the relative expression (ΔCt values) of pairs of genes within each test group. The mean SD (mSD) was used to assess the expression stability of the 13 candidate genes (Supplementary Table S1). A low mSD value indicates stable expression. In all samples and in the NaCl subset, *PP2A* was the most stable gene, whereas *GAPDH* was the least stable gene. *ACT* was the most stable gene in PEG subset and *CYP2* was the most stable gene in MeJA subset, whereas *β-TUB* was the least stable gene in both subsets. In the ABA subset, *EXP2* and *PP2A* were the top two stable genes, whereas *EF1-α* was the least stable gene. Across all organs, *EXP1* and *ACT* were the most stable genes and *GAPDH* was the least stable gene.

**Figure 2:**
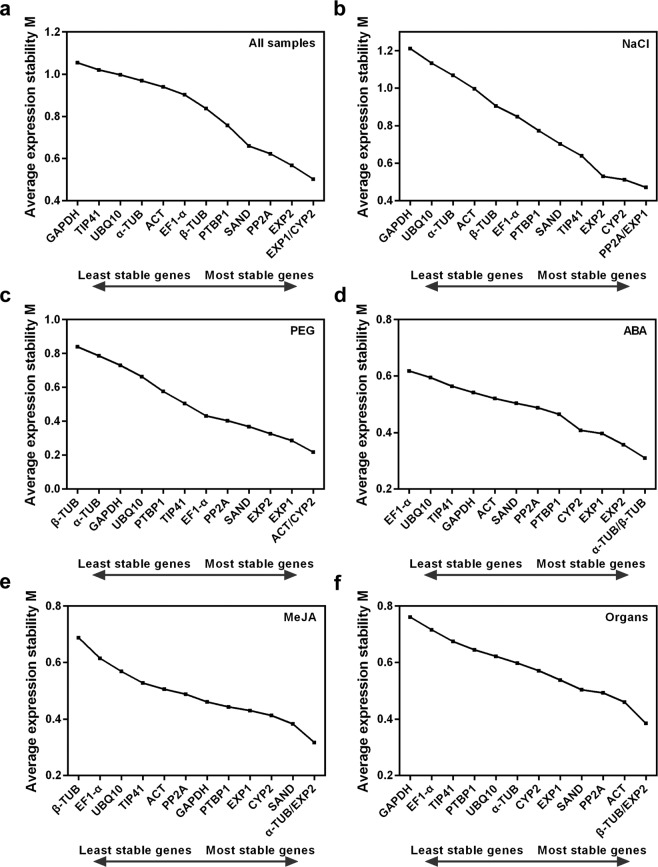
Expression stability and ranking of the 13 reference genes by geNorm. The least stable genes are on the left, and the most stable on the right.

The geNorm algorithm is used to rank the stability of reference genes by generating expression stability values (M-values); a low M-value indicates stable expression (Fig. 2). *EXP1* and *CYP2* were the most stable genes in all test samples, *PP2A* and *EXP1* were most stable in NaCl subset. *ACT* and *CYP2* were most stable in the PEG subset. Equally, *α-TUB* and *β-TUB* in the ABA subset, *α-TUB* and *EXP2* in the MeJA subset, and *β-TUB* and *EXP2* in the organs were the most stable genes. Across all test samples, *GAPDH* was the least stable gene, as in the NaCl and organ subsets. *β-TUB* was the least stable gene in the PEG and MeJA subsets. In the ABA subset, *EF1-α* was the least stable gene.

According to the NormFinder algorithm, genes with the lowest stability values (SV) have the most stable expression. The NormFinder ranked list of the 13 candidate genes was shown in Supplementary Table S2. *PP2A* was the most stable gene in all test samples, followed by *EXP2*, *CYP2*, and *PTBP1*. In the NaCl subset, *PTBP1* was the most stable gene, followed by *CYP2*, *PP2A*, and *EXP1*. In the PEG subset, *PP2A* ranked first, followed by *EXP1*, *CYP2*, and *ACT*. In the ABA subset, *EXP2* was the most stable gene, followed by *PP2A, PTBP1* and *CYP2*. In the MeJA subset, *CYP2* was the most stable gene. Across all organs, *EXP1* and *ACT* was the top two stable reference genes.

The Bestkeeper algorithm calculates the stability of candidate reference genes based on the coefficient of variance (CV) and SD values. The reference gene with the lowest CV ± SD was considered the most stable gene. The BestKeeper ranked list of the 13 candidate reference genes can be found in Supplementary Table S3. In all test samples, *CYP2* (which had the lowest CV ± SD value) exhibited stability levels similar to that calculated by geNorm. In the NaCl subset, *EXP2* was the most stable gene. In the PEG subset, *ACT* was the most stable gene. In the ABA and MeJA subsets, *PP2A* was the most stable gene. Across all organs, *EF1-α* was the reference gene most suitable for normalisation.

**Figure 3:**
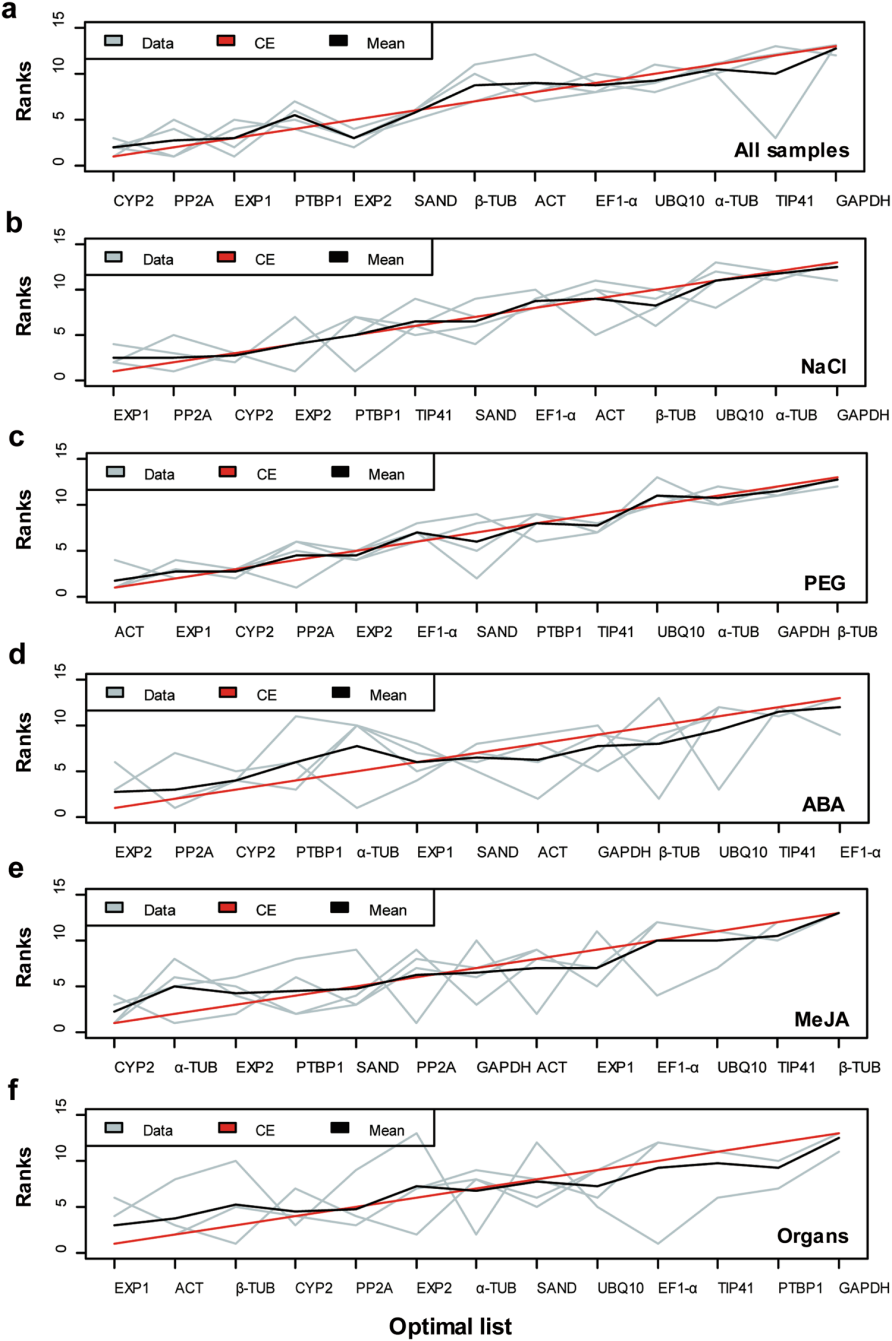
Rank aggregation of the 13 candidate reference genes in the six subsets. The RankAggreg package was loaded into R software. The ΔCt, BestKeeper, NormFinder, and geNorm ranks are represented as grey lines. The black line represents the mean rank of each gene according to each method. The red line indicates the result of the Cross-Entropy algorithm.

The use of different algorithms resulted in different gene expression ranks. Therefore, the ranks of the candidate reference genes were determined using the R package RankAggreg^25^. RankAggreg aggregated the ranks determined by ΔCt, geNorm, NormFinder, and BestKeeper to produce a comprehensive ranking list. For all test samples, the stability of the candidate reference genes was in the order *CYP2* > *PP2A* > *EXP1* > *PTBP1* > *EXP2* > *SAND* > *β-TUB* > *ACT* > *EF1-α* > *UBQ10* > *α-TUB* > *TIP41* > *GAPDH* (Fig. 3a). *EXP1* comprehensively ranked first in the NaCl and organ subsets, and *ACT* and *EXP1* were ranked as the top two stable genes in the PEG subset (Fig. 3b,c,f). In the ABA and MeJA subsets, *EXP2* and *CYP2* were the most stably expressed genes, respectively (Fig. 3d,e). The expression of *GAPDH* was most unstable in the NaCl and organ subsets (Fig. 3b,f). The expression of *β-TUB* was most unstable in response to PEG and MeJA (Fig. 3c,e). For the ABA subset, *EF1-α* was the least stable gene (Fig. 3d).

### Selection of the optimal combination of PCR reference genes

**Figure 4:**
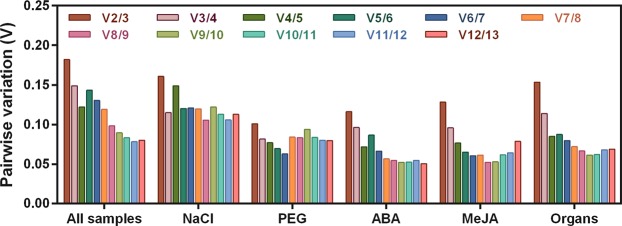
Pairwise variation (V_n_/V_n+1_) values calculated by geNorm to determine the optimal number of reference genes. The cut-off value to determine the optimal number of reference genes for normalisation using qRT-PCR was 0.15.

Using the geNorm algorithm, the pairwise variation V_n_/V_n+1_ was also automatically calculated by geNorm between two consecutive normalisation factors across subsets to determine the optimal number of reference genes^22^. A value of V_n_/V_n+1_ of <0.15 indicated that n reference genes were the optimal combination compared with n + 1 reference genes. Figure 4 shows that all pairwise variations of the PEG, ABA, and MeJA subsets were <0.15, suggesting that the use of two reference genes was the optimal combination for normalising gene expression. In all test samples, and in the NaCl and organ subsets, the V_3_/V_4_ values were <0.15; thus, in these subsets, the use of three reference genes was the optimal combination for normalisation (Fig. 4). For example, according to the comprehensive ranking list (Fig. 3), “*ACT* + *EXP1*”, “*EXP2* + *PP2A*” and “*CYP2* + *α-TUB*” could be regarded as the optimal combinations of qRT-PCR reference genes for the PEG, ABA and MeJA subsets, respectively. Likewise, “*CYP2* + *EXP1* + *PP2A*” could be regarded as the optimal combination of qRT-PCR reference genes for all test samples and the NaCl subset. “*EXP1* + *ACT* + *β-TUB*” was regarded as the optimal combination for the organ subset.

### Validation of reference genes

**Figure 5:**
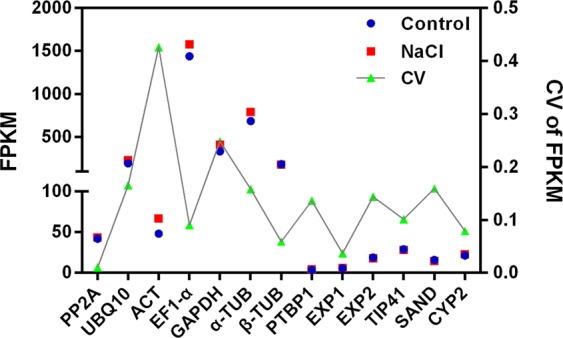
Expression and stability of the candidate reference genes based on RNA-seq in the salt (NaCl) treatment. FPKM values indicate gene expression. Lower coefficient of variation (CV) values indicate more stable expression. FPKM, fragments per kilobase of exon model per million mapped reads.

To validate the stability of the candidate reference genes, we first compared their expression in the presence and absence of NaCl stress based on the RNA-seq analysis of *G*. *littoralis* in response to salt stress^2^. Gene expression levels are reported as FPKM values, and the variation in expression levels as CVs. A lower CV indicates more stable expression. *PP2A*, *EXP1*, *CYP2*, and *β-TUB* had lower CVs than the other candidate reference genes under NaCl treatment, whereas *UBQ10*, *α-TUB, GAPDH*, and *ACT* had higher CVs (Fig. 5). The stability of these genes in the RNA-seq data was mostly consistent with the comprehensive list in the NaCl-treatment subset (Fig. 3b).

**Figure 6:**
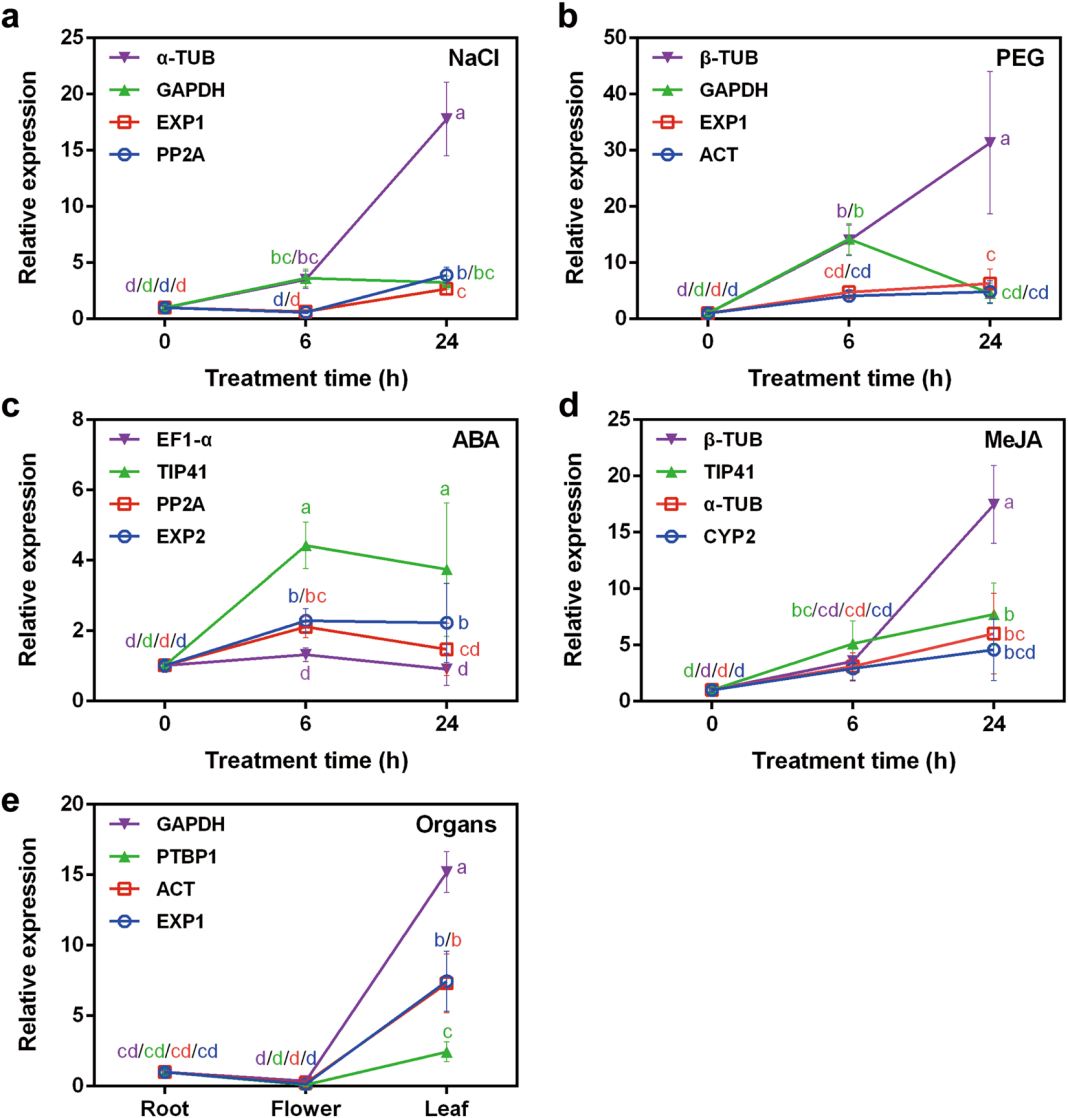
Relative expression levels of *PYL* normalised to four reference genes for NaCl, PEG, ABA, MeJA and organs subsets. Error bars indicate means ± SD. Different letters indicate statistically significant difference in each condition (P < 0.05, Duncan’s multiple range test).

To further validate the reference genes, the expression pattern of a stress-related target gene, *PYL* (Pyrabactin resistance 1-like gene), in the NaCl, PEG, ABA, MeJA, and organ subsets was examined using qRT-PCR (Fig. 6). The two most (rank1 and 2) and least (rank 12 and 13) stable reference genes in Fig. 3 were used as the reference genes for each subset. Different letters indicate statistically significant difference in each condition (P < 0.05, Duncan’s multiple range test). *PYL* expression increased significantly at 24 h after NaCl treatment when normalised to the four candidate reference genes, but the expression was markedly higher when normalised to *α-TUB*. At 6 h, the *PYL* expression showed the opposite trend and significant difference when normalised to *GAPDH* and *α-TUB* (least stable) compared to *PP2A* and *EXP1* (most stable) (Fig. 6a). In the PEG subset, the expression levels of *PYL* after PEG treatment were upregulated when normalised to *GAPDH* and *β-TUB* (least stable) or to *EXP1* and *ACT* (most stable). At 6 h, *PYL* expression was significantly upregulated when normalised to *GAPDH* and *β-TUB* compared to *EXP1* and *ACT*, and *PYL* expression showed the opposite trend at 24 h after PEG treatment when normalised to *GAPDH* compared to the other three candidate reference genes (Fig. 6b). In the ABA subset, *PYL* expression was upregulated at 6 h and decreased at 24 h when normalised to *EF1-α, TIP41* (both least stable), *PP2A*, and *EXP2* (both most stable) (Fig. 6c). However, the *PYL* expression showed no statistical significant compared with that at time 0 h when normalised to *EF1-α*. In the MeJA subset, the variation in *PYL* expression normalised to *CYP2*, *α-TUB* (both most stable), *β-TUB*, and *TIP41* (both least stable) increased at 6 h and 24 h after treatment, and the expression level of *PYL* was more variable when normalised to *β-TUB* (Fig. 6d). The expression level of *PYL* was highest in leaves followed by roots when normalised to the most stable (*EXP1* and *ACT*) and least stable (*GAPDH* and *PTBP1*) genes (Fig. 6e). Therefore, when the most stable reference gene was used for normalisation, the expression level of target gene was more reliable and reproducible.

**Figure 7:**
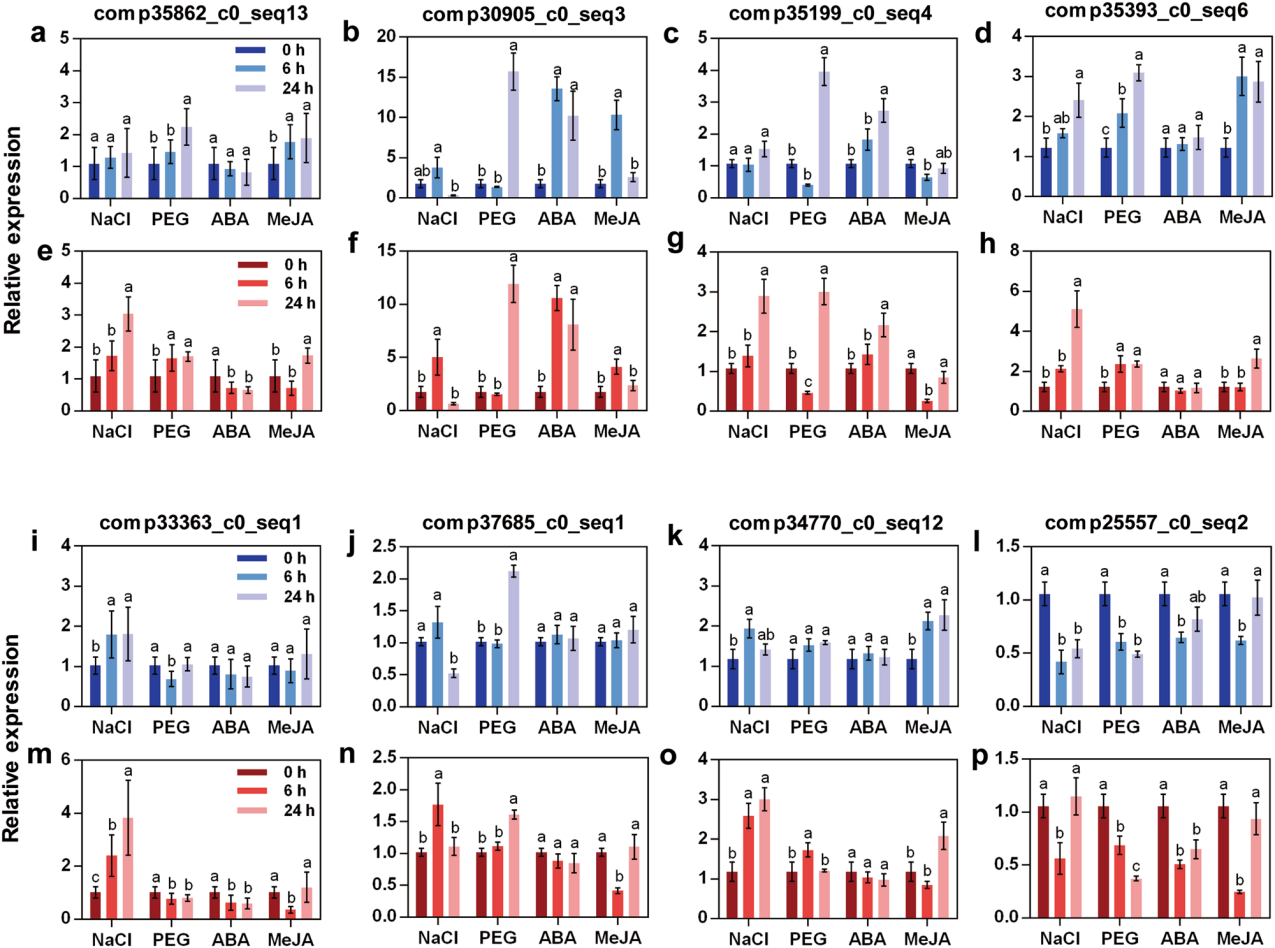
Expression patterns of eight stress-related genes normalised to the top two stable reference genes for NaCl, PEG, ABA, MeJA treatment. *EXP1, ACT, EXP2*, and *CYP2* were used for NaCl, PEG, ABA, MeJA treatments respectively in (**a**–**d**,**i**–**l**). *PP2A, EXP1, PP2A*, and *α-TUB* were used for each treatment respectively in (**e**–**h**,**m**–**p**). (**a**,**e**) comp35862_c0_seq13/MAPK; (**b**,**f**) comp34770_c0_seq12/CDPK; (**c**,**g**) comp30905_c0_seq3/SOS1; (**d**,**h**) comp35199_c0_seq4/CIPK; (**i**,**m**) comp35393_c0_seq6/TPC1; (**j**,**n**) comp25557_c0_seq2/WRKY; (**k**,**o**) comp33363_c0_seq1/P5CS; (**l, p**) comp37685_c0_seq1/SnRK2. Error bars indicate means ± SD. Different letters indicate statistically significant difference in each treatment (P < 0.05, Duncan’s multiple range test).

We then selected eight potentially stress-related genes from *G*. *littoralis* RNA-seq data for expression analysis using the top two stable reference genes of each stress treatment. The eight genes included stress-responding protein kinases, transporter proteins, a transcription factor and a synthetase (Fig. 7; Supplementary Table S4). Reference genes *EXP1, ACT, EXP2*, and *CYP2* were used for NaCl, PEG, ABA, and MeJA treatments respectively in Fig. 7a–d,i–l. *PP2A, EXP1, PP2A*, and *α-TUB* were also used for each treatment respectively in Fig. 7e–h,m–p. Different letters above each bar indicate statistically significant difference in each treatment (P < 0.05, Duncan’s multiple range test). The eight selected genes showed differential expression under NaCl, PEG, ABA and MeJA treatments by qRT-PCR; most were induced by these treatments (Fig. 7). The expression levels of comp35393_c0_seq6, comp33363_c0_seq1, and comp34770_c0_seq12 were significantly increased after NaCl treatment when normalised to both stable reference genes. The expression of comp35862_c0_seq13, comp30905_c0_seq3, comp35199_c0_seq4, comp35393_c0_seq6, comp37685_c0_seq1, and comp25557_c0_seq2 showed significant differences at 24 h after PEG treatment compared with that at 0 h. The expression of comp30905_c0_seq3 and comp35199_c0_seq4 were significantly increased after ABA treatment. The expression of comp35862_c0_seq13, comp35393_c0_seq6, and comp34770_c0_seq12 showed significant differences at 24 h after MeJA treatment compared with that at 0 h, as well as comp30905_c0_seq3 and comp25557_c0_seq2 at 6 h. Although the fold changes in relative expression normalised to the top two stable reference genes did not match perfectly, the trends in expression of these genes were generally consistent, confirming the reproducibility of the qRT-PCR results.

## Discussion

*Glehnia littoralis* is an important medicinal halophyte used in studies of the salt-tolerance mechanism, breeding, synthesis of active ingredients, and the relationship between high salinity and metabolism in *G. littoralis*. Additionally, qRT-PCR is a highly sensitive, accurate, and high-throughput gene expression analysis technology^26^. However, the accuracy of qRT-PCR depends largely on the reference genes used. It is particularly important to select appropriate reference genes for the tissue, organ, and experimental conditions^27^. At present, the use and selection of reference genes, and the stability of these genes are rarely verified in studies of *G. littoralis*, which hampers research. We used the RNA-seq data of *G. littoralis* to screen 13 candidate reference genes. The expression levels and stability of the 13 candidate reference genes were assessed in roots of *G. littoralis* subjected to NaCl, PEG, ABA, and MeJA treatments, as well as in the major organs. *G. littoralis* is a halophyte and its leaves have the structure typical of most mesophytes^28^. Plant hormones regulate all aspects of plant growth and development. MeJA, a derivative of jasmonate, is an important signalling molecule in plant secondary metabolism^29–31^. We ranked the stability of the 13 candidate reference genes under a variety of conditions using the ΔCt, geNorm, NormFinder, and BestKeeper algorithms. Because of their different principles, the rank lists generated by the four algorithms were non-identical (Fig. 2; Supplementary Tables S1–S3)^32^. For example, *PP2A* was ranked first by geNorm and ΔCt in the NaCl subset, but it was ranked third by Normfinder and fifth by BestKeeper. The geNorm algorithm calculates the SD of variation between two reference genes after logarithmic transformation as the M-value and indicates the optimal number of reference genes necessary for accurate normalisation^22^. Normfinder works on a similar principle as geNorm, but it considers both the inter- and intra-group variation in expression and outputs only the most stable gene^23^. BestKeeper, an Excel-based tool, identifies stable reference genes using pair-wise correlation and can analyse up to 10 target genes^24,33,34^. The ΔCt method determines the stability of reference genes by comparing the relative expression (Ct value) of pairs of genes within each sample based on geNorm data^21^. In the present study, in the NaCl and PEG subsets, the gene ranked first by geNorm was identical to that ranked by the ΔCt method. For all samples and in the ABA, MeJA and organ subsets, the gene ranked first by Normfinder was identical to that ranked by the ΔCt method. Although the stability ranks of the candidate reference genes differed among the four methods, the RankAggreg R package or a geometric mean analysis can be used to combine the rank lists into an optimal list of reference genes^25,35,36^. Several previous studies have suggested that the use of a combination of reference genes is better than a single reference gene^36–38^. The geNorm algorithm also determined the optimal number of reference genes for accurate normalisation by pairwise variation between the normalisation factors. For different cases, an optimal combination of reference genes can be used for comprehensive normalisation to acquire the most reasonable results. In the present study, the use of two reference genes was the optimal combination for qRT-PCR analysis under PEG, ABA and MeJA treatments, and the use of three reference genes was the best for all test samples, NaCl and organ subsets.

Housekeeping genes with stable constitutive expression, such as *ACT, TUB, 18S rRNA, UBQ, CYP, GAPDH*, and *EF1-*α, are typically used as reference genes. However, the expression levels of these reference genes are not constant in some species and cases^16,18,39–42^. *GAPDH* has good stability in *Arabidopsis pumilain* under drought- and heat-stress and in Kentucky bluegrass roots under cold- and drought-stress but was one of the most unstable reference genes in *Baphicacanthus cusia*^43^ as well as in *G. littoralis* in this study. Similarly, *β-TUB* was the least stable reference gene in the roots of *G. littoralis* exposed to PEG and MeJA, as well as in *Metasequoia* exposed to ABA^36^. However, *β-TUB* showed good stability in *Syntrichia caninervis* under abiotic stresses and desiccation/rehydration^44^, and in a variety of tissues of *Corchorus capsularis*^32^. In this study, *CYP2* was highly stable in *G. littoralis* under salt stress but was less stable in orchardgrass^45^. Novel reference genes, such as *PP2A*, *EXP1*, *TIP41*, and *SAND*, exhibited better stability in specific cases^18,19,46,47^. For example, *PP2A* and *SAND* were highly stable in *Caragana intermedia* under salt-, drought-, and heat-stress^47^, and *EXP1* was the most stable gene in *Lycoris aurea* under salt-, heat-, and ABA-stress and in various tissues^35^. In this study, *PP2A*, *EXP1*, *EXP2*, and *CYP2* were the four most stable genes in *G. littoralis* in most subsets. In *Peucedanum praeruptorum* Dunn, a species in Umbelliferae, *SAND*, *ACT2*, *UBC9*, *PP2A*, and *PTBP1* were the most suitable genes for normalisation of qRT-PCR data^48^.

In our previous RNA-seq analysis of *G. littoralis*, we also assessed differentially expressed genes (DEGs) under salt stress. The results of the previous study were compared against the stability assessment in the present study for the NaCl treatment to confirm the stability and reliability of the candidate reference genes (Figs. 3 and 5). To validate the stability of these reference genes, *PYL* was first used as a test gene (Fig. 6). *PYL* is a core regulatory component of the ABA signaling pathway, and its expression is induced by various stresses^49^. In the transcriptome analysis of *G*. *littoralis* in response to salt stress^2^, *PYL* expression was significantly upregulated by salt stress. In our study, the two most and least stable reference genes were used as the reference genes for each subset. The more stable the reference gene, the more reliable the expression trend of the target gene. For example, a clearly opposite trend in *PYL* expression was observed at 24 h when *GAPDH* (unstable reference gene) was used as the reference gene compared to the most stable reference gene for PEG treatment, as well as when it was used as the reference gene for NaCl treatment, which can lead to misinterpretation of results, as some previous studies have indicated^50–52^. For further validation, we analysed and compared the relative expression levels of other stress-related genes from *G*. *littoralis* RNA-seq data under different stress conditions with the top two stable reference genes of each subset. The qRT-PCR results revealed similar trends in the expression patterns of these genes when the two selected reference genes were used for normalisation. These genes include Ca^2+^-dependent protein kinase (*CDPK*), CBL-interacting protein kinase (*CIPK*), mitogen-activated protein kinase (*MAPK*), and the ser/thr protein kinase OPENSTOMATA 1 *(OST1)/SnRK2.6/SnRK2E*, all of which play pivotal roles in plant responses to multiple stresses^53–56^; salt overly sensitive (SOS) signaling pathway gene *SOS1* and vacuolar ion channel TWO PORE CHANNEL1 (*TPC1*), which show positive responses to salt stress^57,58^; ∆1-pyrroline-5-carboxylate synthetase (*P5CS*), which resists drought stress^59^; and the transcription factor *WRKY*^60^. Most of these genes display stress-induced expression in *G. littoralis* as well as in other plants. All of the above results provide the evidence for validation of the stability of the recommended reference genes. Therefore, the use of appropriate reference genes or gene combinations is essential for normalisation of the expression of target genes in *G. littoralis*. Our systematic analysis of candidate reference genes will facilitate future studies on gene expression in *G. littoralis*, as well as in other species of Umbelliferae.

## Materials and methods

### Plant materials and stress treatments

*Glehnia littoralis* seeds were originally collected from Tannanwan Beach, Pingtan, Fujian Province, China (25°26′1.86″N, 119°45′14.4″E) under the permission of competent authority and cultivated in the Germplasm Garden of Institute of Botany, Jiangsu Province and Chinese Academy of Sciences, Nanjing, China^2^. The *G*. *littoralis* was identified and conserved by the Herbarium of Institute of Botany, Jiangsu Province and Chinese Academy of Sciences (voucher specimen: No.0631502). The experimental research on *G. littoralis* is legal and no field experiments are involved. In this study, seedlings of *G*. *littoralis* with a root length of 4–6 cm were transplanted from Germplasm Garden into flowerpots containing washed sand. The seedlings were grown in a chamber under a 14-h-light (26 °C)/10-h-dark (22 °C) photoperiod and irrigated with Hoagland nutrient solution. After 3 months of growth, the seedlings were subjected to salt (200 mM NaCl), drought (20% PEG 6000), or hormone (100 μM ABA or 100 μM MeJA) treatment for 0, 6, or 24 h, as described previously^35^. The underground part of *G*. *littoralis* is used as herbal medicine, and so *G*. *littoralis* roots were sampled separately at different time points for each treatment. For organ samples, leaves and flowers of *G*. *littoralis* were collected in July during the same year. Plants grown under normal conditions were collected as controls. Samples were harvested from three replicate plants, immediately frozen, and stored at −80 °C for RNA extraction.

### RNA extraction, cDNA synthesis, and qRT-PCR

RNA from *G. littoralis* was extracted using RNAiso Plus reagent (TaKaRa, Dalian, China) according to the manufacturer’s instructions. The RNA concentration was measured using a Nanodrop ND-2000 spectrophotometer (Thermo Scientific, Waltham, MA, USA). RNA samples with a ratio of absorbance at 260 nm (A_260_) to absorbance at 280 nm (A_280_) of 1.8–2.2 and an A_260_/A_230_ ratio of > 1.8 were used for cDNA synthesis. For qRT-PCR, 600 ng of RNA was used for reverse transcription in a 20 μL reaction volume using the Prime Script™ RT Reagent kit with gDNA Eraser (TaKaRa).

The qRT-PCR amplification was performed in 96-well plates using a qTOWER 2.2 Real-Time PCR system (Analytik Jena AG, Jena, Germany) with TB Green Premix Ex Taq™ II (Tli RNaseH Plus; TaKaRa). Reactions were performed in a total volume of 20 μL containing 2.0 μL of 10-fold diluted cDNA, 0.8 μL each of forward and reverse primer (10 μM), 10 μL of TB Green Premix Ex Taq™ II, and 6.4 μL of ddH_2_O. A reaction without template was used as the negative control. The qRT-PCR conditions were as follows: 95 °C for 5 min followed by 40 cycles of 95 °C for 15 s, 60 °C for 15 s, and 72 °C for 25 s. After completion of amplification, a melting curve analysis was performed. Three biological replicates and three technical replicates were performed.

### Selection of candidate reference genes and design of primers

We previously performed a comprehensive transcriptome analysis of *G*. *littoralis* in response to salt stress via Illumina 2000 sequencing^2^. A number of unigenes were identified and annotated, and their expression was analysed. Based on commonly used reference genes in *Arabidopsis*, we identified 13 candidate reference genes from the *G.littoralis* transcriptome data. Primers for qRT-PCR were designed based on the sequences of the 13 candidate reference genes (Supplementary File S1) using Primer3 Tools (http://primer3.ut.ee/) and the following criteria: primer size 18–23 bp, GC content 45–65%, melting temperature 57–62 °C, and product size 100–250 bp (Table 1). The specificity of the primer pairs was assessed via qRT-PCR and a melting curve analysis. The *G*. *littoralis* cDNA template was diluted five-fold with nuclease-free water to establish a calibration curve. The PCR amplification efficiency (*E*) and the correlation coefficient (R^2^) were calculated using the slope of the calibration curve according to the equation 10^−1/slope^ – 1^61^. Finally, the amplification products were verified via 1% agarose gel electrophoresis and sequencing.

### Analysis of gene stability

The stability of the 13 candidate reference genes was evaluated using four statistical algorithms—the ΔCt method^21^, geNorm^22^, NormFinder^23^, and BestKeeper^24^. The ΔCT method involved comparison of the relative expression of pairs of genes within each sample. NormFinder, geNorm, and BestKeeper are Excel-based tools, and the Ct values obtained were converted into input files according to the developers’ instructions^35,36,44^. The geNorm algorithms also recommended the optimal combination number of qRT-PCR reference genes by comparing pairwise variation (V_n_/_n+1_) values and the cut-off value was <0.15^22^. Using the above four algorithms, ranked lists of gene stability were generated, and the R package RankAggreg was used to generate a list of the overall stability of the 13 candidate reference genes^25^. R software v. 3.6.0 (R × 64) was used to load the RankAggreg v. 0.6.5 package (http://cran.r-project.org/web/packages/RankAggreg/). The details can be accessed at https://cran.r-project.org/web/packages/RankAggreg/RankAggreg.pdf. The RankAggreg package contains a Cross-Entropy Monte Carlo algorithm and a Genetic algorithm for evaluating rank aggregation.

### Validation of the reference genes

Several stress-related genes were selected from the *G*. *littoralis* transcriptome data to test the stability of the candidate reference genes. The details of them were shown in Supplementary Table S4. Primers for qRT-PCR were shown in Supplementary Table S5. Relative gene expression was analysed using the 2^−ΔΔCT^ method^62^.

## Supplementary information

Supplementary Information.

Dataset 1.

## Data Availability

The authors confirm that the data supporting the findings of this study are available within the article and its Supplementary Information.
