# Supplementary material for: Selection and Validation of Reference Genes for Normalisation of Gene Expression in Glehnia littoralis
Source: Sci Rep. 2020 Apr 30;10:7374. doi: 10.1038/s41598-020-63917-5 (PMC7192926; doi:10.1038/s41598-020-63917-5)

Selection and Validation of Reference Genes for Normalisation of Gene Expression in *Glehnia littoralis*

Li Li, Naiwei Li, Hailing Fang, Xiwu Qi and Yifeng Zhou *

Supplementary Information

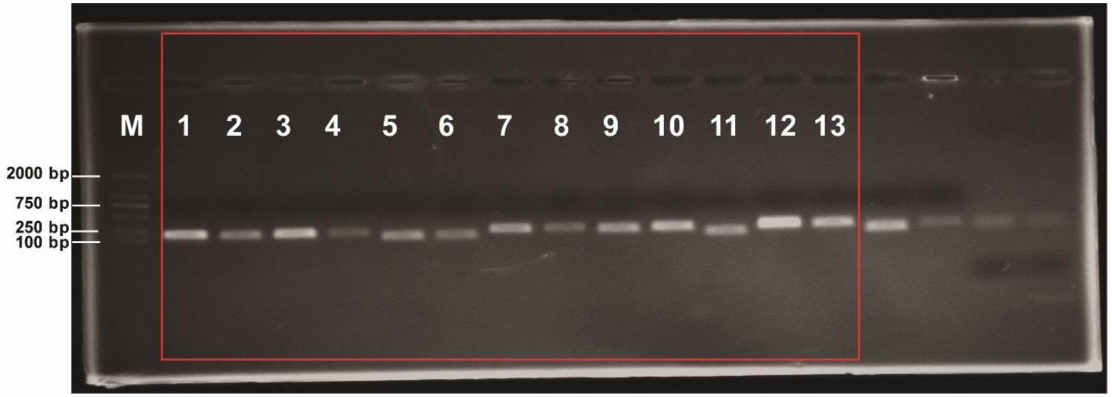

Supplementary Figure S1. Agarose gel (1%) electrophoresis for the qRT-PCR amplicons of 13 candidate reference genes using qRT-PCR primers. Here provides a full-length gel. The qRT-PCR amplicons of 13 candidate reference genes are shown in the red rectangle. The M represents DNA marker having bands of 2000 bp, 1000 bp, 750 bp, 500 bp, 250 bp and 100 bp. 1–13 represent *PP2A*, *UBQ10*, *ACT, EF1-α*, *GAPDH*, *α-TUB*, *β-TUB*, *PTBP1*, *EXP1*, *EXP2*, *TIP41*, *SAND*, *and CYP2,* respectively.

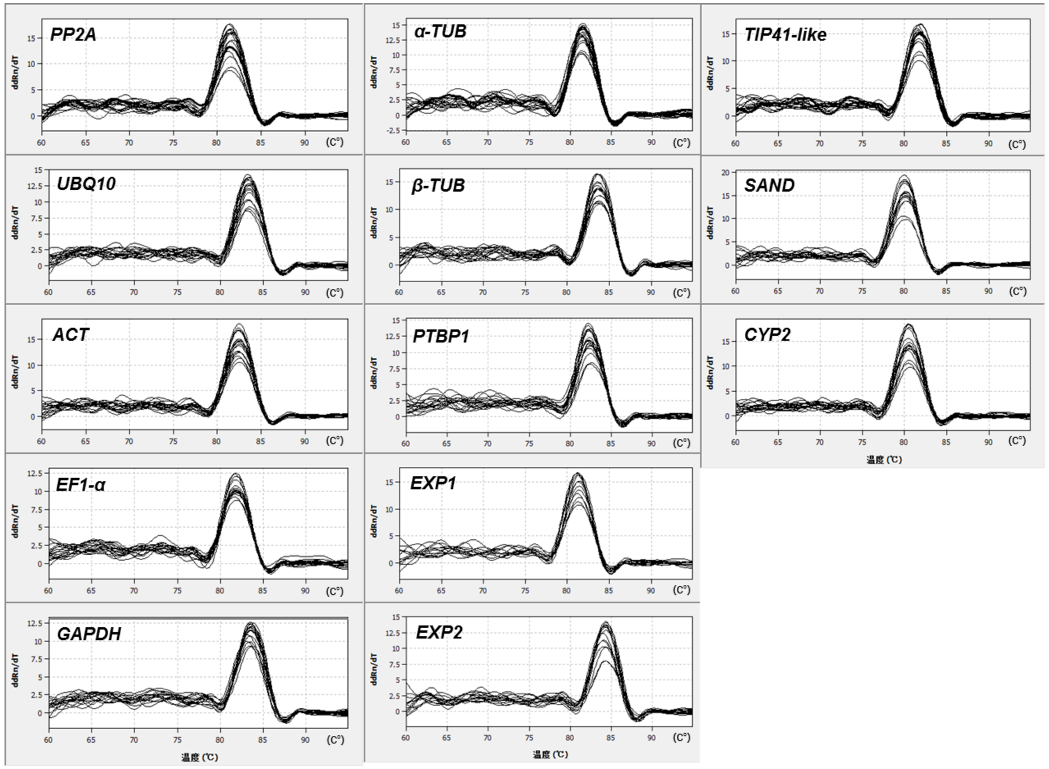

Supplementary Figure S2. Melting curves of 13 candidate reference genes in this study.

Supplementary Table S1. Candidate reference gene ranking based on ΔCt method.

Rank	All samples	mSD	NaCl	mSD	PEG	mSD	ABA	mSD	MeJA	mSD	Organs	mSD
1	*PP2A*	0.892	*PP2A*	0.998	*ACT*	0.647	*EXP2*	0.522	*CYP2*	0.530	*EXP1*	0.642
2	*CYP2*	0.919	*EXP1*	0.999	*EXP1*	0.649	*PP2A*	0.536	*PTBP1*	0.558	*ACT*	0.652
3	*EXP2*	0.935	*CYP2*	1.031	*CYP2*	0.650	*CYP2*	0.538	*SAND*	0.587	*PP2A*	0.661
4	*EXP1*	0.941	*EXP2*	1.041	*EXP2*	0.688	*PTBP1*	0.568	*EXP2*	0.606	*CYP2*	0.666
5	*PTBP1*	1.000	*PTBP1*	1.065	*PP2A*	0.692	*EXP1*	0.583	*EXP1*	0.610	*β-TUB*	0.693
6	*SAND*	1.018	*β-TUB*	1.126	*EF1-α*	0.776	*ACT*	0.592	*GAPDH*	0.625	*SAND*	0.725
7	*β-TUB*	1.092	*SAND*	1.205	*TIP41*	0.789	*SAND*	0.592	*PP2A*	0.627	*EXP2*	0.727
8	*ACT*	1.093	*EF1-α*	1.214	*SAND*	0.802	*β-TUB*	0.598	*α-TUB*	0.627	*α-TUB*	0.759
9	*UBQ10*	1.116	*TIP41*	1.242	*PTBP1*	0.894	*GAPDH*	0.637	*ACT*	0.658	*UBQ10*	0.760
10	*EF1-α*	1.132	*ACT*	1.306	*α-TUB*	1.007	*α-TUB*	0.647	*TIP41*	0.721	*PTBP1*	0.796
11	*α-TUB*	1.147	*UBQ10*	1.426	*UBQ10*	1.094	*TIP41*	0.735	*UBQ10*	0.815	*TIP41*	0.884
12	*TIP41*	1.184	*α-TUB*	1.464	*GAPDH*	1.095	*UBQ10*	0.741	*EF1-α*	0.894	*EF1-α*	0.922
13	*GAPDH*	1.242	*GAPDH*	1.639	*β-TUB*	1.131	*EF1-α*	0.748	*β-TUB*	1.090	*GAPDH*	1.007

Supplementary Table S2. NormFinder-based expression stability evaluation of 13 candidate reference genes.

Rank	All samples	Stability value	NaCl	Stability value	PEG	Stability value	ABA	Stability value	MeJA	Stability value	Organs	Stability value
1	*PP2A*	0.289	*PTBP1*	0.294	*PP2A*	0.160	*EXP2*	0.151	*CYP2*	0.067	*EXP1*	0.225
2	*EXP2*	0.342	*CYP2*	0.357	*EXP1*	0.181	*PP2A*	0.169	*PTBP1*	0.104	*ACT*	0.231
3	*CYP2*	0.355	*PP2A*	0.368	*CYP2*	0.218	*PTBP1*	0.198	*GAPDH*	0.167	*PP2A*	0.234
4	*PTBP1*	0.361	*EXP1*	0.370	*ACT*	0.223	*CYP2*	0.202	*SAND*	0.179	*CYP2*	0.249
5	*EXP1*	0.367	*ACT*	0.426	*EXP2*	0.231	*GAPDH*	0.231	*EXP2*	0.185	*β-TUB*	0.276
6	*SAND*	0.437	*TIP41*	0.435	*PTBP1*	0.252	*SAND*	0.243	*α-TUB*	0.213	*UBQ10*	0.306
7	*ACT*	0.467	*EXP2*	0.438	*TIP41*	0.267	*EXP1*	0.243	*EXP1*	0.249	*EXP2*	0.323
8	*EF1-α*	0.479	*β-TUB*	0.455	*EF1-α*	0.293	*ACT*	0.248	*ACT*	0.254	*SAND*	0.333
9	*UBQ10*	0.502	*SAND*	0.493	*SAND*	0.400	*β-TUB*	0.251	*PP2A*	0.272	*α-TUB*	0.333
10	*β-TUB*	0.503	*EF1-α*	0.503	*α-TUB*	0.515	*α-TUB*	0.281	*TIP41*	0.308	*PTBP1*	0.349
11	*α-TUB*	0.516	*GAPDH*	0.697	*GAPDH*	0.569	*UBQ10*	0.314	*UBQ10*	0.411	*TIP41*	0.443
12	*GAPDH*	0.560	*α-TUB*	0.709	*β-TUB*	0.578	*TIP41*	0.354	*EF1-α*	0.462	*EF1-α*	0.458
13	*TIP41*	0.574	*UBQ10*	0.727	*UBQ10*	0.601	*EF1-α*	0.359	*β-TUB*	0.632	*GAPDH*	0.519

Supplementary Table S3. BestKeeper-based expression stability evaluation of 13 candidate reference genes.

Rank	All samples	CV	SD	NaCl	CV	SD	PEG	CV	SD	ABA	CV	SD	MeJA	CV	SD	Organs	CV	SD
1	*CYP2*	2.83	0.69	*EXP2*	3.24	0.80	*ACT*	1.65	0.34	*PP2A*	1.50	0.34	*PP2A*	1.99	0.45	*EF1-α*	4.73	0.92
2	*EXP1*	2.81	0.74	*EXP1*	3.25	0.85	*SAND*	1.43	0.34	*ACT*	1.66	0.34	*ACT*	2.25	0.47	*α-TUB*	4.76	0.98
3	*TIP41*	3.20	0.77	*CYP2*	3.50	0.87	*CYP2*	1.44	0.34	*UBQ10*	1.76	0.39	*CYP2*	2.17	0.52	*CYP2*	4.21	1.02
4	*EXP2*	3.44	0.85	*SAND*	3.58	0.90	*EXP1*	1.36	0.35	*CYP2*	1.69	0.41	*EF1-α*	3.00	0.55	*EXP1*	4.17	1.10
5	*PP2A*	3.74	0.86	*PP2A*	4.08	0.96	*EXP2*	1.55	0.37	*SAND*	1.72	0.42	*α-TUB*	2.87	0.58	*UBQ10*	5.09	1.14
6	*SAND*	4.06	1.01	*TIP41*	4.48	1.08	*PP2A*	1.91	0.44	*EXP2*	1.76	0.42	*EXP2*	2.50	0.61	*TIP41*	4.99	1.15
7	*PTBP1*	4.05	1.03	*PTBP1*	4.97	1.30	*EF1-α*	2.45	0.46	*GAPDH*	2.65	0.52	*UBQ10*	2.91	0.64	*PTBP1*	5.43	1.35
8	*UBQ10*	4.79	1.08	*UBQ10*	6.56	1.52	*PTBP1*	2.38	0.60	*EXP1*	2.03	0.52	*PTBP1*	2.67	0.67	*ACT*	6.29	1.37
9	*EF1-α*	5.91	1.13	*EF1-α*	7.19	1.45	*TIP41*	2.54	0.60	*EF1-α*	2.99	0.54	*SAND*	2.80	0.70	*PP2A*	6.13	1.43
10	*α-TUB*	5.66	1.21	*β-TUB*	7.59	1.66	*UBQ10*	3.53	0.78	*α-TUB*	2.67	0.54	*GAPDH*	3.50	0.70	*β-TUB*	7.18	1.49
11	*β-TUB*	5.83	1.25	*ACT*	8.56	1.93	*α-TUB*	4.66	0.98	*PTBP1*	2.22	0.54	*EXP1*	2.81	0.73	*GAPDH*	7.15	1.51
12	*ACT*	5.85	1.25	*α-TUB*	9.52	2.12	*GAPDH*	5.15	1.03	*TIP41*	2.45	0.58	*TIP41*	3.08	0.73	*SAND*	6.05	1.52
13	*GAPDH*	6.21	1.29	*GAPDH*	9.69	2.05	*β-TUB*	5.10	1.08	*β-TUB*	3.04	0.61	*β-TUB*	4.49	0.93	*EXP2*	6.19	1.54

Supplementary Table S4. Information of stress-related genes in *G. littoralis* transcriptome.

**Gene_id**	**NR_tophit_name**	**Description**
comp10674_c0_seq1	gi|460390986|ref|XP_004241105.1|	PYL
comp35862_c0_seq13	gi|297748058|gb|ADI52619.1|	MAPK
comp34770_c0_seq12	gi|2827773|sp|P28582.2|CDPK_DAUCA	CDPK
comp30905_c0_seq3	gi|225424514|ref|XP_002285248.1|	SOS1
comp35199_c0_seq4	gi|508782317|gb|EOY29573.1|	CIPK
comp35393_c0_seq6	gi|470134518|ref|XP_004303095.1|	TPC1
comp25557_c0_seq2	gi|350540824|gb|AEQ29025.1|	WRKY
comp33363_c0_seq1	gi|6225816|sp|O04015.1|P5CS_ACTDE	P5CS
comp37685_c0_seq1	gi|502143007|ref|XP_004505181.1|	SnRK2

Supplementary Table S5. qRT-PCR primers of stress-related genes used in this study.

Primer	Sequence (5' to 3')
PYL-F	TGCCTTCATCTCTTCGACGT
PYL-R	CATGTGTGTGTGGTGGTGTT
MAPK-F	GTGGTTTCGATGTGGCCATT
MAPK-R	ACTTCGGCATGGCAAAACAT
CDPK-F	ATGGATGAGAGAAGGCGGAG
CDPK-R	TACCACTCTTGTCCGTGTCC
SOS1-F	CTGATCCTGTTGCTGTCGTG
SOS1-R	ATTTGACAACAGCAGCCCAG
CIPK-F	CACTGCCTTTCACAACACCA
CIPK-R	TTACTTGCCGGTCATTTGCC
TPC1-F	ACGCTTGCCAATTTCCCATT
TPC1-R	AGATCTTTGGCGGGTTGGTA
WRKY-F	TGGAGAGGTGCTTGGAAGAG
WRKY-R	GCCTTCCTCTTAACGCCTCT
P5CS-F	GTTGGCATCAGTACGAGCAG
P5CS-R	AAGCCTTCTGAGTGAGGTCC
SnRK2-F	GGCCCGGTTGATGAGAGATA
SnRK2-R	TTCGAACAGCTCTCCTCCAG

Supplementary File S1. Sequences of candidate reference genes from *G. littoralis* transcriptome.

PP2A

ATGTCGAATGTTGAAGAGCCATTGTACCCAATAGCTGTGTTGATTGATGAGCTGAAGAATGACGATATTCAGTTGCGACTTAATTCTATTAG
AAGGCTTTCGACTATTGCTCGTGCTCTCGGGGAGGAACGGACAAGAAGGGAATTGATTCCGTTTTTGAGCGAGAACAATGATGATGACGAT
GAGGTTCTTCTTGCAATGGCCGAAGAGTTGGGAGTATTTATTCCTTATGTTGGAGGTGTGGAACACGCTCATGTTTTGCTCCCCACTCTTGA
AAATCTTTGCACTGTTGAAGAAACTTGTGTCAGGGACAAAGCCGTGGAGTCGCTTTGTAGAATTGGATCTCAGATGAGGGAGAGTGATTTA
AATGACTATTTTGTTCCTCTAGTGAAGAGGTTGGCAGCAGGTGAATGGTTTACTGCTCGAGTTTCTGCTTGTGGATTGTTTCACATCGCATA
TCCCAGTGCACCAGAGACGTTGAAAACCGAATTGCGATCGATATACAGTCAGTTGTGTCAGGATGACATGCCTATGGTTAGGAGATCTGCT
GCTACAAATTTGGGGAAGTTTGCTGCAACCATTGAACCTGCTCATCTCAAGACTGATATTATGTCAATATTTGAGGATCTTACCCAGGATGA
TCAAGATTCTGTTCGGTTGCTAGCTGTTGAAGGATGTGCTGCTCTTGGAAAGTTGTTGGAACCTCAAGATTGTGTAGCACATATACTTCCCG
TTATTGTCAACTTTTCCCAGGATAAGTCGTGGCGTGTTCGTTACATGGTTGCCAATCAGCTCTATGAATTATGTGAAGCTGTGGGACCTGAA
CCTACCAGGACGGAATTAGTTCCTGCATATGTGCGACTTCTTCGAGATAATGAAGCCGAAGTACGTATAGCTGCTGCTGGCAAAGTCACCA
AGTTCTGCAGAATTCTTAATCCTGAGCTAGCAATTCAGCACATCCTTCCATGTGTGAAGGAGTTATCATCTGATTCTTCTCAGCATGTTAGAT
CTGCTCTGGCCTCGGTTATAATGGGCATGGCTCCTGTATTAGGAAAGGAAGCAACAATCGAGCAGCTTCTTCCAATATTTCTTTCCCTTCTG
AAGGATGAATTTCCTGATGTGCGACTCAACATTATTAGCAAGCTAGATCAAGTTAATCAGGTTATTGGAATAGATCTTTTGTCCCAGTCTCTG
CTACCAGCCATTGTTGAGCTTGCAGGGGATAGGCATTGGAGAGTTCGACTAGCAATTATTGAGTATATTCCGCTATTAGCTAGCCAATTAGG
AGTAGGCTTCTTCGATGATAAGCTTGGTACTCTCTGTATGGAGTGGCTAAAGGATAAGGTTTGCTCGATTCGAGATGCTGCTGCTGATAACT
TGAAGCGCCTTGCAGAAGAATTTGGCCCAGAGTGGGCGATGCAGCATATCATTCCACAGGTGTTGGACATGATTAATAACCCTCATTACCT
GTATCGGATGACAATCCTGCGTGCTATATCTCTACTTGCTCCTGTCATGGGCCCAGAAATCACATGTTCTAAACTGCTACCTGTTCTAGTTA
CTGCATCAAAGGACAGAGTGGCGAACATCAAATTCAATGTGGCAAAGGTGCTGCAATCCCTTATTACTGTAGTCGATCAGTCTGTGGTGGA

GTCAACAATTCGCCCTTGTCTGGTGGAACTTAGCGAGGACCCGGATGTCGATGTTCGTTTCTTTGCCAATGAAGCACTTCATGCTATTGATC
ATGACATGATGTCAAGCTAG

UBQ10

ATGCAAATTTTTGTCAAGACCCTCACTGGGAAGACGATTACCTTGGAAGTAGAGAGCTCGGATACAATTGACAATGTGAAGGCGAAAATTC
AAGACAAGGAAGGAATCCCTCCTGATCAGCAAAGGTTGATATTTGCTGGCAAGCAGTTAGAGGATGGAAGGACTTTGGCCGATTACAATAT
TCAGAAGGAATCAACCCTTCATTTGGTGCTGAGGCTGAGGGGTGGAATGCAGATTTTTGTGAAGACTTTGACGGGGAAAACCATCACCCTG
GAGGTGGAGAGCTCGGACACCATTGATAATGTCAAAGCAAAAATACAGGACAAAGAAGGTATCCCACCAGACCAGCAGAGGCTGATTTTT
GCTGGCAAGCAGCTTGAGGATGGTCGTACACTTGCAGACTACAACATCCAAAAGGAATCTACCCTTCACTTGGTGCTACGTCTCAGAGGAG
GGATGCAGATCTTTGTCAAGACTTTGACTGGTAAGACCATTACTCTGGAGGTTGAAAGCTCGGATACCATTGATAATGTGAAGGCAAAGAT
CCAAGACAAGGAGGGAATCCCACCAGATCAGCAGAGGTTGATTTTTGCTGGAAAGCAGTTGGAAGAT

ACT

ATGGCCGATGCTGAGGATATCCAGCCCCTAGTTTGTGACAATGGAACTGGAATGGTGAAGGCTGGTTTTGCTGGTGATGATGCTCCCAGA
GCAGTATTCCCCAGTATTGTTGGTAGGCCCAGACATACTGGTGTTATGGTCGGGATGGGGCAGAAGGATGCCTATGTTGGTGATGAAGCC
CAATCGAAGAGAGGTATTCTTACCTTGAAATATCCGATTGAGCACGGTATTGTGAGTAATTGGGATGACATGGAGAAAATTTGGCATCATAC
CTTTTACAATGAGCTTCGAGTTGCTCCTGAGGAGCACCCAGTTCTTTTGACTGAAGCGCCTCTCAATCCCAAGGCCAACAGGGAGAAAATG
ACTCAGATTATGTTTGAGACGTTTAATGTTCCTGCTATGTATGTTGCCATCCAGGCTGTTCTTTCTCTGTATGCAAGTGGTCGTACTACTGGT
ATTGTGCTGGATTCTGGTGATGGTGTGAGCCATACTGTACCAATTTACGAAGGATATGCCCTTCCCCATGCCATTCTCCGTCTCGACCTTGC
TGGTCGTGATCTCACTGATTCTCTCATGAAGATCTTAACGGAGAGAGGTTACATGTTCACCACCACTGCTGAGCGGGAAATTGTTCGTGAC
ATGAAGGAGAAACTTGCCTATGTTGCTCTTGACTACGAGCAAGAGCTTGAAACCTCAAAGAGTAGCTCTTCTGTGGAAAAGAACTATGAATT
GCCTGACGGACAAGTTATTACAATTGGAGCTGAGAGATTCCGTTGCCCAGAAGTCCTGTTCCAGCCGTCTCTGATCGGGATGGAAGCTGC
TGGAATCCATGAAACCACTTACAACTCCATCATGAAGTGTGATGTCGATATCAGAAAGGATCTCTATGGAAACATAGTGCTCAGTGGTGGTT
CAACAATGTTCCCCGGTATTGCAGATCGTATGAGCAAGGAAATTACTGCCCTTGCACCCAGCAGCATGAAGATCAAAGTTGTTGCACCACC

CGAGAGAAAATACAGTGTCTGGATTGGAGGATCCATTCTTGCATCTCTCAGCACCTTCCAACAGATGTGGATATCCAAGGGCGAATATGAC
GAGTCTGGCCCATCAATCGTGCACAGGAAGTGTTTTTAA

EF1α

ATGGGTAAGGAAAAGATTCATATCAGTATTGTGGTCATTGGCCATGTCGACTCTGGAAAGTCTACCACAACTGGTCATCTTATCTACAAGCT
AGGTGGTATCGACAAGCGTGTGATTGAAAGGTTCGAGAAGGAAGCTGCTGAGATGAACAAACGTTCATTCAAGTACGCATGGGTTCTTGAC
AAGCTTAAGGCTGAGCGTGAACGTGGTATTACCATTGATATTGCTCTTTGGAAGTTTGAGACTACCAAGTACTACTGCACAGTTATTGATGC
TCCAGGGCATCGTGATTTCATTAAGAACATGATTACTGGAACTTCTCAGGCTGATTGTGCTGTCCTGATCATTGACTCCACCACTGGAGGTT
TTGAAGCTGGTATCTCTAAGGATGGGCAAACTCGTGAGCATGCTTTGCTTGCATTTACACTTGGTGTCAAGCAGATGATCTGTTGCTGCAAC
AAGATGGATGCTACAACCCCCAAGTACTCCAAGTCTAGATTCGAAGAAATTGTGAAGGAGGTTTCTTCTTATTTGAAGAAGGTTGGGTACAA
CCCCGACAAAATTGCTTTCATTCCCATCTCTGGATTTGAGGGTGACAACATGATTGATAGGTCTACCAACCTTGACTGGTACAAGGGACCAA
CTCTTCTTGAAGCTCTTGACCAGATCTCTGAGCCCAAGAGACCCTCAGACAAGCCCCTTCGTCTCCCACTTCAGGATGTTTACAAGATTGG
AGGTATTGGAACTGTGCCAGTGGGACGTGTTGAAACTGGTGTGATCAAGCCTGGTATGGTTGTGACTTTTGGTCCTTCAGGGTTGACCACT
GAAGTCAAGTCTGTTGAGATGCATCATGAGGCTCTCCAGGAGGCTCTTCCTGGTGACAATGTTGGATTCAATGTTAAGAATGTTGCTGTTAA
GGATCTCAAGCGTGGATATGTTGCCTCCAACTCCAAGGATGATCCCGCCAAAGAGGCTGCCAATTTCACTGCTCAAGTTATCATCATGAAC
CACCCTGGTCAGATCTCAAATGGTTATGCTCCAGTGCTTGATTGCCATACCTGTCACATTGCTGTTAAGTTTGCTGAAATCCAAACCAAGAT
TGATCGTCGATCTGGTAAGGAGATCGAGAAGGAGCCCAAGTTTTTGAAGAATGGTGATGCTGGATTTGTTAAGATGATTCCAACCAAGCCC
ATGGTGGTCGAGACCTTTATGACCTACCCTCCTCTTGGAAGGTTTGCTGTAAGGGACATGAGGCAGACTGTTGCTGTGGGAGTCATCAAGA
GTGTGGAGAAGAAGGAACCTACCGGAGCCAAGGTCACAAAGGCGGCAATCAAGAAGAAA

GAPDH

ATGGCACCAATCAAGATCGGAATCAACGGTTTCGGAAGAATTGGACGATTGGTTGCTAGAGTTGTTCTGCAAAGAGATGATGTTGAGCTTG
TTGCTGTTAACGATCCATTTATCTCAACTGATTACATGACATACATGTTCAAGTATGACAGTGTTCACGGTGCATGGAAGCATCATGAACTCA
AGGTTAAGGATGAGAAGACTCTTCTCTTCGGTGCGAAGCCTGTTGCTGTCTTTGGTTGCAGGAACCCAGAGGAGATCCCATGGGCTAGCA
CTGGTGCAGAGTATATTGTTGAATCCACTGGTGTCTTCACTGACAAGGAAAAGGCTGCTGCACATTTGAAGGGAGGTGCAAAGAAGGTCAT

CATATCTGCCCCAAGCAAAGATGCTCCAATGTTTGTCGTTGGTGTCAATGAGAAGGAATACAAGTCTGACCTCCACATTGTTTCCAATGCTA
GTTGCACAACAAATTGCCTTGCTCCCCTAGCTAAGGTGATCAATGATAGGTTTGGCATTGTTGAGGGGCTTATGACAACTGTTCATTCAATC
ACTGCCACACAAAAAACTGTTGACGGACCTTCTGCGAAGGACTGGAGAGGTGGAAGAGCTGCTTCATTCAACATCATTCCTAGCAGCACTG
GAGCTGCCAAGGCTGTTGGAAAAGTGCTACCTTCTCTGAATGGGAAGTTGACCGGAATGTCATTCCGAGTTCCTACTGTGGATGTCTCAGT
TGTTGATCTCACTGTCAGGCTGGAAAAGAAGGCTACTTATGAACAAATTAAAGCTGCCATTAAGGAGGAGTCTGAGGGAAAGCTTAAGGGA
ATCTTGGGTTACACTGAAGATGATGTGGTTTCCACAGACTTTGTGGGTGACAGCAGGTCAAGCATCTTTGATGCCAAAGCTGGAATTGCTC
TAAATGACAACTTTGTCAAGCTTGTTTCGTGGTATGACAACGAATGGGGATACAGCACCCGAGTGGTTGACTTGATCGTTCATATGGCATCT
GTTCA

α-TUB

ATGAGGGAGTGCATTTCAGTTCACATCGGTCAGGCCGGTATTCAGATCGGTAACGCTTGCTGGGAACTTTACTGCCTCGAGCACGGCATTC
AGCCTGATGGCCAAATGCCAAGTGACAAAACTGTCGGTGGAGGTGATGATGCTTTCAACACTTTCTTTAGTGAAACTGGTGCTGGAAAGCA
TGTGCCTCGAGCAATCTTTGTGGATCTTGAGCCCACTGTCATTGATGAAGTGAGGACTGGAACATATCGTCAGCTCTTTCATCCTGAACAG
CTGATTAGCGGAAAAGAAGATGCAGCTAACAACTTTGCTCGTGGACACTATACCATTGGAAAGGAGATTGTTGATCTTTGCCTGGATCGTAT
CAGGAAGCTTGCTGACAATTGCACTGGTCTCCAGGGTTTCCTTGTTTTTAATGCTGTTGGAGGAGGCACTGGTTCTGGTTTGGGTTCCCTT
CTTCTGGAACGTCTCTCCGTGGACTATGGCAAAAAGTCAAAACTTGGATTCACTGTTTATCCTTCACCACAGATCTCTACCTCTGTTGTTGA
GCCTTACAACAGTGTGCTTTCGACCCACTCACTTTTGGAGCACACCGATGTTTCTGTGCTGCTGGATAATGAGGCTATATATGATATTTGCA
AGCGTTCCCTTGACATTGAGCGACCCACCTATACCAACCTTAATCGATTGGTTTCTCAGGTCATTTCCTCTTTGACCGCTTCCTTGAGGTTT
GATGGAGCCTTGAATGTTGATGTGACTGAGTTCCAGACTAATCTGGTGCCATACCCAAGGATCCACTTCATGCTTTCTTCTTATGCCCCTGT
TATCTCCGCTGAGAAGGCCTACCATGAACAGCTATCTGTTGCAGAGATCACCAACAGTGCATTTGAGCCCTCTTCTATGATGGCCAAGTGT
GATCCTCGCCATGGAAAGTACATGGCTTGCTGTCTGATGTACCGAGGTGATGTGGTGCCCAAAGATGTGAATGCAGCTGTTGGTACCATTA
AGACCAAGCGCACCATCCAGTTTGTTGATTGGTGCCCAACTGGTTTTAAGTGCGGTATCAACTATCAGGCCCCAACTGTTGTTCCAGGTGG
TGATCTTGCCAAAGTGCAGAGAGCTGTATGCATGATCTCAAATTCGACCAGTGTTGCAGAGGTTTTCTCACGCATAGACACTAAATTTGACC

TAATGTACTCAAAGAGGGCTTTCGTTCACTGGTATGTTGGCGAGGGTATGGAAGAAGGTGAATTCTCTGAAGCACGTGAGGATCTTGCTGC
CCTTGAGAAGGACTATGAGGAGGTTGGTGCAGAGTCTGCTGAGGGGGATGATGAGGACGAGGGAGAAGATTACTGA

β-TUB

ATGAGAGAAATTCTTCACATTCAGGGCGGTCAATGTGGAAACCAGATCGGAGCAAAGTTCTGGGAAGTGATCTGCGCCGAGCACGGGATC
GATTCGACAGGGCGTTACCAGGGAGACACTGAAATTCAATTGGAGCGAATCAATGTGTATTACAATGAAGCCAGTTCTCAGAGGTATGTTC
CCAGGGCTGTGCTTATGGATCTGGAGCCTGGTACTATGGATAGTCTCCGATCTGGACCCTACGGTCAGATCTTCAGGCCTGATAACTTTGT
GTTTGGTCAATCTGGTGCTGGTAATAATTGGGCCAAAGGTCACTATACTGAAGGTGCTGAGTTAATCGACTCGGTGCTTGATGTTGTGAGG
AAGGAAGCTGAGAATTGTGACTGTCTTCAAGGGTTTCAGGTGTGTCATTCACTCGGTGGTGGAACCGGATCTGGAATGGGTACACTTCTGA
TTTCAAAAATCAGAGAGGAGTATCCTGACCGTATGATGCTTACTTTCTCAGTTTTCCCATCACCCAAGGTGTCTGATACTGTGGTTGAGCCT
TATAATGCCACTCTTTCTGTTCATCAACTTGTTGAAAATGCTGATGAGTGCATGGTTTTGGACAATGAGGCTCTTTATGACATTTGCTTCCGC
ACCTTGAAGCTTACCACACCTAGCTTTGGTGATCTAAACCACTTGATTTCGGCCACTATGTCTGGTGTTACATGCTGCTTGCGTTTCCCTGG
TCAGTTGAACTCCGATCTCAGGAAGTTGGCTGTAAATCTCATTCCCTTCCCCAGGTTGCACTTCTTTATGGTTGGATTTGCACCTCTTACCT
CCCGTGGTTCCCAGCAATACCGTGCATTGAGTGTACCTGAGCTTACCCAGCAGATGTGGGATTCAAAGAACATGATGTGCGCAGCTGATC
CCCGCCATGGTAGATACTTGACAGCTTCTGCTGTGTTCAGAGGAAAGATGAGCACTAAAGAGGTTGATGAGCAGATGATCAACGTCCAGAA
CAAGAACTCTTCCTACTTTGTTGAATGGATCCCAAACAATGTGAAGTCAACTGTTTGTGACATCCCACCAACTGGTCTGAAGATGGCTTCAA
CCTTCATTGGGAATTCAACTTCAATTCAAGAGATGTTTAGGCGTGTGAGTGAGCAGTTCACAGCTATGTTCAGGAGGAAAGCTTTCTTGCAT
TGGTATACCGGTGAGGGCATGGACGAGATGGAGTTCACTGAGGCTGAGAGCAACATGAATGATCTTGTTTCGGAGTACCAGCAGTACCAG
GATGCCACTGCTGACGAGGAGGGTGACTATTTCGAAGAAGAAGAAGAGGATGGCCAAGACATGTAA

PTBP1

ATGTCGAATTCAAATCAGCCTCAATTTCGATACACACAGACTCCTTCTAAAGTGCTTCACTTGCGTAACTTGCCTTGGGAGTGTATTGAAGA
AGAGCTCGTCGAGCTTTGCAGGCCTTTTGGTAAGATCGTTAACACCAAGTGCAATGTCGGCGCTAATCGCAATCAAGCCTTCGTTGAATTT
GTGGATCTTAATCAGGCCATTAATATGGTTTCATATTATGCTTCATCATCAGAACCTGCATCTGTTCGGGGTAAACATGTTTATATACAGTAT
TCAAACAGACATGAAATTGTCAACAACAAGGGTCCAGGTGATGTTCCGGGAAATGTCTTGCTGGTAACCATTGAGGGTGTAGAAGCCGGTG

ATGTAAGCATTGATGTGATTCACTTGGTCTTCTCGGCTTTTGGATTTGTGCACAAGATTGCTACTTTTGAGAAGGCAGCAGGTTTTCAGGCA
CTAATCCAGTTTACTGATGCTGAGACTGCTCTTTCAGCAAGGGAAGCTTTAGATGGCAGAAGTATTCCAAGGTACTTGCTTCCAGAACATGT
TGGTTCTTGCAATCTGCGCATCTCATATTCAGCTCACACAGATCTAAACATCAAGTTCCAATCACACCGTAGCCGGGACTATACAAATCCAT
ATCTTCCTGTTAATGCAACTGCAATTGAGGGATTTGTCCAGCCTGTTGTAGGTCCTGACGGAAAGAAAAAAGAACCGGAGAGTAATGTACTT
CTTGCCTCAATTGAAAATAGGATCTATGATGTCACTGTAGATGTTCTTAACACGGTATTCTCTGCATTTGGCACGGTTCAGAAAATTGCTATA
TTCGAGAAGAACGCGACAACTCAGGCTCTAATTCAGTATCCTGATGTCAACACTGCCGCCGTAGCTAAAGATGCTCTAGAGGGACACTGCA
TATATGATGGTGGCTACTGTAAGCTTCATATATCATACTCTCGTCATACTGATCTCAATGTAAAGGCCTTCAGCGATAAAAGTAGGGATTATA
CAGTACCAGAGTCCGGTTTTGCTGCTGGTCTGCCTGCTGGAGCAACAGTCTGGCAGAATCCTCATGCTGCTGCTCCGGTCTTTATTGGGA
GCGAATTTGCTAGTATCAATTATGGGCAGCCTCAAGGCTCTCCCGGTCAAGGACCTCCTGG

EXP1

ATGTCGAAAACTGAAGACGAAGAGGAGCGCCGGAGAAAGTACGAGGAAGCTCTCGAAGTCAAATCTCTCCGCCGTATCATCAGCGCCTAT
CTCAATTACCCAGAGGCTGCAGAGGAGGACTTGAAAAGATATGAAAGATCTTATAGAAGGCTTCCACCAACCCATAAGGGTCTCCTGTCTC
ACCTTCCTGTAAAATATCGAAGACTGCGAAGGTGTATATCTAAGAATTCATATTTTATATTTGAAATGCTAAAGGCATTTGAACCTCCCCTTG
ATCTGAGCCAAGACCTTGACATATGTGAACAAGATCCGCAGAATATCTTAGACGATACCAAAGAAACCAATTATTTTTCTTGTGGGTCTGCA
TCAACCAGTAAAACAGGATGTCATCCAGGGTGCAATGAAGCTGTCAGTGGAGAGGAGGGGAGCGTGTTATTAGGATCTCCCAAGGAGGAG
AAACTTGGGCTTTTTATTGATTCGGACACCGGGAGCCGTCATATTTTGGAATGTGATGCCACAGCAGATAAAGCTGGTAACAACGGTGTTA
AGATTAAAAAAACTTCACACTCTAATGCAGACTCCAATAATAATGAGAAACTTGGGCTTTTCATTGAGTCTGACACTGGGAACCGTCATGTTT
TGGAATGTGATACCAAAGCAGATGAGGCTGGTAACAACGGTGTTAGGATACAGGAAACTTCGTACTCTAATGCAGACTCCAATTATAATGT
GTCTTCATCTCCTGATTGGTTGGATCCATCACTGCAGTCGCATGTTCCTCTAGTTGATGTAGATAAGGTTCGATGTATTATAAGAAATATTGT
AAGAGATTGGGCTGCAGAGGGACAACAAGAACGTGATCAGTGCTATACGCCTATTCTTGAAGAGCTTAAATCACAATTTCCTAATCGAAGTA
AAGGGAGCCCTCCTGCATGTTTAGTTCCGGGTGCTGGACTTGGTAGACTGGCTTTGGAAATTTCATGTCTTGGTTTTGCAAGCCAAGGAAA
TGAATTTTCATACTATATGATGATCTGCTCGAGTTTTATTCTTAACCAAGCGGAAAGGGCTAATGAATGGACTATCCATCCTTGGATTCATAG
CAATTGCAATTCACTTTCTGACAGTGACCAGCTTCGTCCTATTTCAATACCAGATATTCATCCTGCCAGGAATAACTGA

EXP2

ATGGCAAGAGGAGAGTGGGGGTACTATAATGGAAGAACGAAATGGTGTTCTTATAGAAGAACCACTTTGATTATTTGTTCAATTAACATTGG
TGTTGCTCTTTATGTTCTTCACACTCTTTATAACTCTCTTTACACCTACCCTTTTAATGATCCTCAAAAAGCTGCTAGGTACACTCCTGATCAG
ATTAGGAAAATGGAAGAATCAAATGATATTAGAAAAGCCTCACAACCCACTGAACTTATTAAATTGGTGAATGAAATAAGGAAGGATTTTTTA
CAAGAAGAGAAGAGGGTTGATTTGCCATCAAATTTGAAACACCAGGTAATTGATGAGATTGTGGAATTATTGAGGAGCTTGAAGTCCTCCAA
TGCGACTGTTCAAAATGAAGCAGTTGAAAGATGGCGCAAGCAAAAAATAAGAGAAGCTAGAGGGGTGGCTCGGGGAGATATTCTGAATCC
AAACATTCTGCCAAAGGAAGCAAAAATTCTTGCAAGAACGTTGAAGTCTCGCTGGGATGAGTTTAGAGAAGAAATCGGTCTCTGGATACCT
GTTGCAATCGTTAACAAGGAACATGATGACAAGCCTGAGGGTGAAGAAGAGTTTGACAGCGAAATATTAGCCGGCAGACAGCTTCCTCCC
GAGTGCCATACTGAACTTCATACAGATTATGGTGGGGCAGCTGTTCGCTGGGGCCTTACCCACCATAAAGAGAGCGCTTATGATTGTTGTC
AAGCTTGTCTGGATCAAGCCAAAAATGCAAGAGAAGGCGAAAAGCGCTGCAATATATGGGTGTACTGCCCTTCAGAGGGTGGATGTTACTC
ACCAGATATATATGAACACAAACAGCAAGAATGCTGGCTGAAATATGACGAGAAACCCCAAGTAAGCTTTAAGGACAAATACTCCGAATCAT
TCAGAAACTCGCATCCAAATGTTCCACTGGTTGTTCCATGGGTAGCTGGGATTGTAAGTGTATAA

TIP41

ATGGTTTTTGGGGAAAGTTCATTGGTTCTCAAGCACTTGAAGAGCGATGTAAAGATTCATTTCAACGCATTTGATTCTCTAGTTGGTTGGAA
GCAGGAAAAATTACCACCAGTTGAGGTCCCTGCAGCAGCAAAATGGAAATTTAGAAGCAAACCTTTCCAGCAGGTGATATTAGATTATGACT
ACACATTTACAACACCATATTGTGGAAGTGAAACTGTTGAGAAAAACTCAGAGAGGGATACAATCTCTGATGAAGGCAGTTGCAAGCTTCGT
TGGGAGGACTGCGAGGAACGAATTAATTTGACTGCACTTGCATCAAAAGAGCCTATTCTCTTCTATGATGAGGTGATCTTCTATGAAGATGA
ATTGGCTGATAGTGGAGTGTCGCTTTTAACAGTAAAAGTGAGAGTGATGCCAAGCTGTTGGTTTCTTCTCTTGCGTTTTTGGCTTAGAGTTG
ATGGTGTGCTTATGCGTTTAAGGGACACACGCATCCATTGCATTTTTGGTGAGGGTAAAACACCAGTTATTCTGAGAGAATGTTGCTGGAGA
GAGGCCACATTTCAAGCACTAGCTTCTAAAGGATATCCTTCTGATTGTGCTGCGTATATTGATCCAAGCAGCATCGGCCAAAGACTTCCTAT
CATTTTGCATAAGACCCAAAAGCTTATAATTCCTGATTAA

SAND

ATGTTACCAGAAGATGATGCCAACTCCTCATCAGAAACCGACTCAATTGACCAAAACCCTAACCCTACCACTTCAATTGACCAATCTCTCGA
CGCTATTGAAGGTCAATTAACCTCTATTTCACTCAATCACCACCACTCAAAACCCCCATTTCACCCTCCTCTTCCCCAAAATATCGATACATT
GCCTTCCCATTCGCATTCGCAACTCCAACAACCACCAGCACCAGCTGAAAATCTCCAAAATATCGATACATTACCTTCCCATTCACATTCGA
AACTCCAACAAGTAGCAGTAGCTGAAAATATCGGTTCATTACCTTCGGATTCACATTCGCGGGCCGGACAAGTAGTTGAAAATTCTGGACA
AGTGGATATATTAGGTTCGGATTCCTATACGAAAGTAGAGAAGGAAGTAGTTGGAAATTCGAGAGGCGAAGGAGTGTTGTGGAGGAATAAT
TCGGATGTGGAAGTTGAGGTGGAAGGGCAAGGGAGTCCGAGTAGTAGTGGATATGCTGGAGGAAAGGGGACTAGTAGTAGTGGTAGTAG
TGGTATAAGTGGTTCAGGTATCGAGGAGATTAGTGGCGGCGATGACGAGGTGGTTAACAGGAGTGGTTCTTTTGGTGGTAGTGTGGATTC
CGAGTGGGTCCCTGGGAAACGGCATGTTAATGAAGATGATGCTTCTGTTTCATGGAGGAAAAGGAAGAAGCATTTTTTTATCTTAAGCCATT
CCGGAAAACCAATATATTCAAGATATGGAGATGAACACAGACTAGCAGGATTTTCAGCAACTTTGCAAGCCATCATTTCCTTCGTGGAGAAT
GGGGGAGATCGCGTGAAGTTGGTTAGGGCGGGAAAACACCAGGTGGTTTTTCTTGTTAAAGGACCAATATATCTAGTTTGCATAAGCTGTA
CAGAAGAGCCTCATGAATCCCTCAGTGAACAACTGGAACTTCTTTATGGCCAGATGATACTTATTCTGACAAAGTCTATAAATAGATGCTTT
GAGAAGAATCCGAAATTTGATATGACACCTTTGCTTGGGGGAACAGATGCTGTGTTCTCTTCTCTCATCCACTCGTTTAGTTGGAACCCTGC
CACTTTTCTTCATGCCTACTCTTGTCTTCCCCTTGCTTATCCAACAAGGCAAGCCGCCGGTGCCATATTGCAGGATGTTGCTGAGTCAGGTG
TCCTCTTCGCGATATTAATGTGTAAACACAAGGTCATCAGTCTGGTTGGTGCACAAAAAGCGTCTCTTCATCCCGATGATATGCTCTTGCTT
GCCAACTTTGTGATGTCATCTGAATCATTCAGGACATCTGAATCTTTCTCTCCAATCTGTCTTCCGAGATACAATCCAATGGCATTTTTATATA
CTTATGTGTATTATCTTGATGCTGATACTTATTTGATGTTGCTTACTGCTAATCCTGATGCATTTCATCGTCTAAAAGATTGGAGGATCCGTAT
CGAAATGGTCCTTCTGAAGTCAAATGTTCTTAATGAAGCTCAAAGGTCGATGTTGGATGGTGGCATGCGTGTCGAAGATGTGCCTGTTAAT
CCATCTCCTCGCTCGGGATCTTTGTCATCTCATTTAGGTCAGCCTAGACCTCCACCGGACTCTGCAGATGGGTGTAAGGCACTGTTAGGTG
GTCCTGCTGGGCTTTGGCACTTCGTTTACCGCAGTATATATCTAGATCAATATGTATCTTCTGAGTTCTCATCACCGATCAACACCCCTAAAC
AACAGAAAAGATTATATAGAGCATATCAGAAGCTGTATACTTCTATGCATGATATAGAACTTGGTCCTCACAAAACCCAGTTTAGAAGGGAC
GAGAACTATGTTCTACTCTGCTGGGTTACTCAGGATTTTGAACTTTATGCAGCATTTGATCCTCTAGCAGACAAGGCACTGGCTATAAAGAC
ATGCAACCGAGTATGCCAATGGGTTAAAGATGTGGAAAATGAAGTTTTTTTGTTGGGAGCAAGCCCCTTTTCATGGTGA

CYP2

ATGTCATCTATCTACGTATCGGAGCCTCCGACCAAAGGCAAAGTTTCGCTCAAGACAACATACGGTCCATTGGACATAGAGCTATGGCCGA
AAGAGGCTCCTAAAGCTGTGCGCAACTTCGTTCAGCTCTGTCTCGAAGGTTATTATGATGACACAATTTTTCATCGTATAATTAAGTCATTTA
TGGTCCAAGGTGGTGATCCTACTGGCACTGGCAAAGGTGGTGAAAGTATATATGGAGGTACATTTTCTGATGAGTTCCATTCCCGCCTTAG
GTTCAACCACAGGGGCTTGGTTGCATGTGCGAATGCTGGATCACCAAATTCAAATGGGAGTCAGTTTTTTATAACCTTGGATCGTTGTGATT
GGCTTGATCGTAAACATACCATTTTCGGAAAGGTAACTGGAGATTCACTATACAATCTCTTAAACTTTTCCGAGGTTGAAACTGATAAGGAT
GATCGACCAGTAGAATCTCCCCCTAAATTGATTTCAGTTGAGGTGATATGGAACCCTTTTGATGATATTGTTCCAAGGGCAGCCCCTGCTAA
AGCTTTGGTCTCCTCAAATGATAGTGGCAACAGAGATACAAAAAGGAAAGCGTCAAAAAAGCTAAACTTGCTTTCATTTGGAGAAGAAGCTG
AAGAAGAGGAAAAAGAATTGGCAGCTGTGAAGATGAAAATTAGAAGTAGTCACGATGTATTAGATGATCCTCGTTTGCTGAAGGAAGACGG
TTCAACCAGCAAACCGAGTGAATCAGAAGCCAAAGCTATGAAAGATATGCAGTTAAGTGTTAGAGAAGCTCTAAGTTCAAAGAAGGATGAAT
CATGGAAAGAGACGCACAGTAAATTTTCAGAGACCCTTCCTGATAGCGATGACGATGAGGCCAACTTTGATAACAGGATGCGATTACAAAT
ACTTAAGAAAAGAAAGGAGCTTGGAGATCATTCAACTAAGCAAAAGTCACACAATGCGAGTTCAAGTCCAAGAAACCGTGAACGCTCCTATT
CTCCTCCCAGGTCAAATGCCAAAAATTCCGATGATCAACCAAAAGTGGAGAAGTTGGCTTTGAAGAAGGGAATAGGATCAGAAGCCAGGG
CCGAGCGTTTGGCCAATGCGGATGTGGACTTGCAACTGTTGGGAGAAGCTGAACGAGAAAGGCAGTTACAAAAGCAGAAGAAGCGCCGA
TGTCATGGGCACGAAGAGGATGTGCTAGCAAAGCTTGAGAAGTTCAAGGCCACCATGTCCTCCAAATCTGTTGGAGCTGATGGTGAATCT
GGAGGACACAAGGAAGAGGACTTGTCTGACTGGACAAAAGTTAAGCTGAAGTTTGAACCTCAATCCGGGAAGGATAATATGACTCGCACC
GAGAATGTGAATGACTATGTATTTCATGATCCTCTTCTGGAGAAGGGAAAAGAGAAGTTCAACAAAATGCAAGCAAAGCAAAAGCGACGAG
AACGAGAATGGGCTGGAAAGTCACTTACATAA

Supplementary File S2. Sanger sequencing results for qRT-PCR amplicons of 13 reference genes.

PP2A

GCAACCATTGAACCTGCTCATCTCAAGACTGATATTATGTCAATATTTGAGGATCTTACCCAGGATGATCAAGATTCTGTTCGGTTGCTAGCT
GTTGAAGGATGTGCTGCTCTTGGAAAGTTGTTGGAACCTCAAGATTGTGTAGCACATATACTTCCCGTTATTGTCAACTTTTCCCAGGATAA
GTCGTGGCGTGTTC

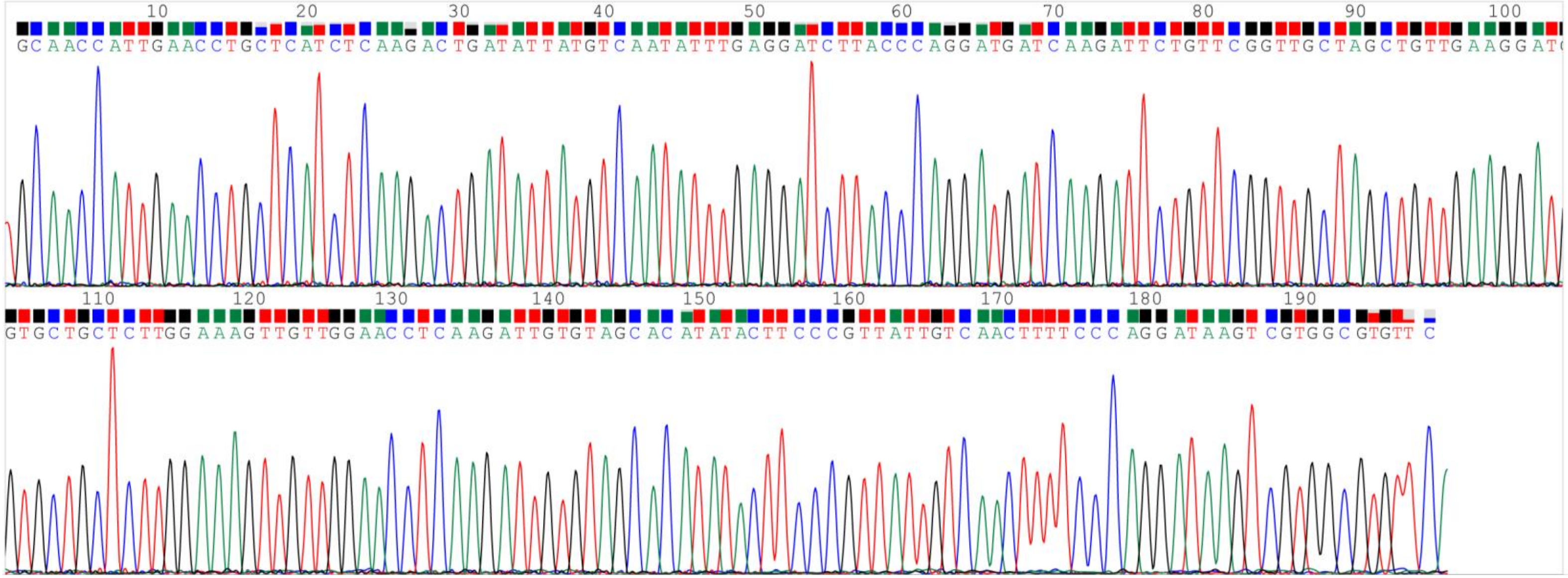

UBQ10

TGAGGGGTGGAATGCAGATTTTTGTGAAGACTTTGACGGGGAAAACCATCACCCTGGAGGTGGAGAGCTCGGACACCATTGATAATGTCA
AAGCAAAAATACAGGACAAAGAAGGTATCCCACCAGACCAGCAGAGGCTGATTTTTGCTGGCAAGCAGCTTGAGGATGGTCGTACACTTG
CA

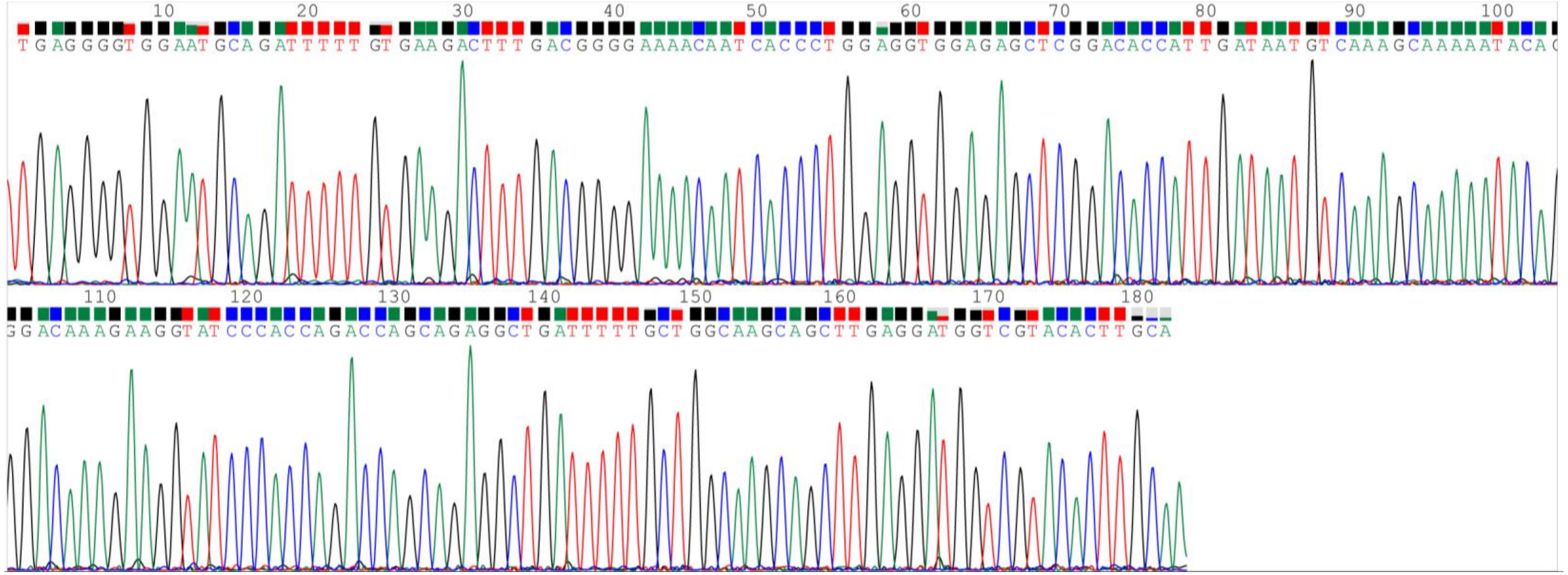

ACT

ACCATCACCAGAATCCAGCACAATACCAGTAGTACGACCACTTGCATACAGAGAAAGAACAGCCTGGATGGCAACATACATAGCAGGAACA
TTAAACGTCTCAAACATAATCTGAGTCATTTTCTCCCTGTTGGCCTTGGGATTGAGAGGCGCTTCAGTCAAAAGAACTGGGTGCTCCTCAGG
AGCAACTCGAAG

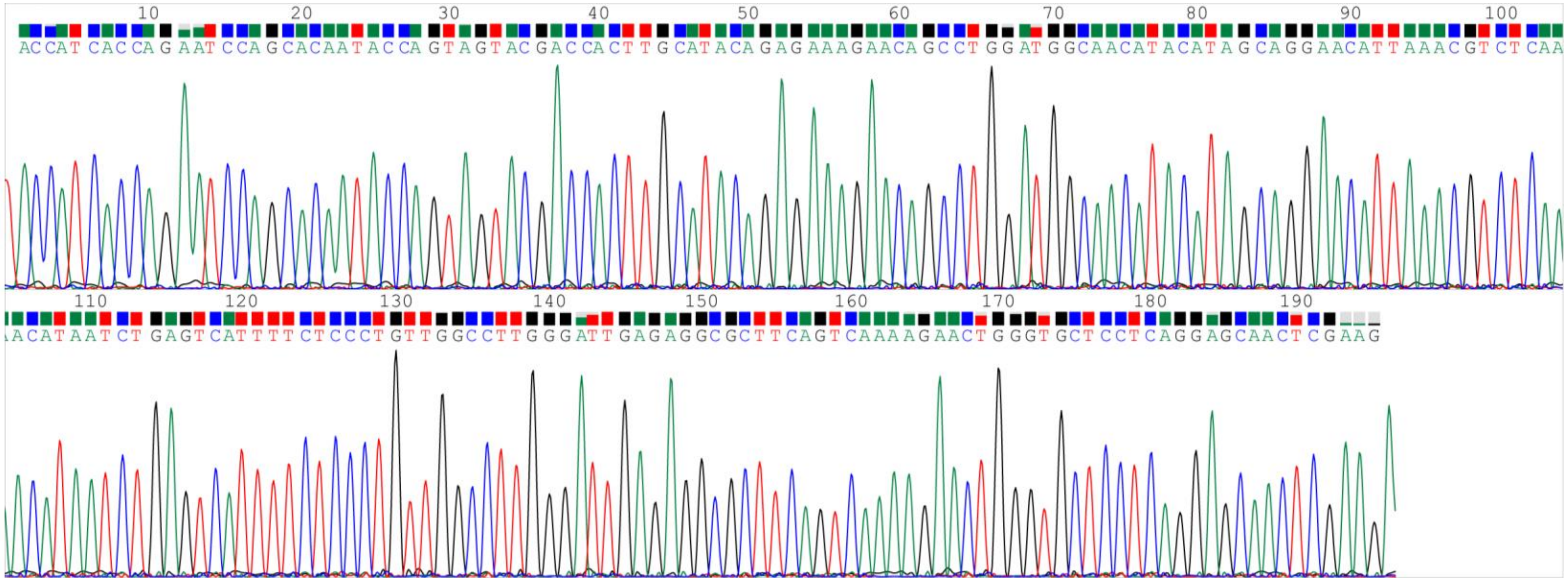

EF1α

AAGGATGGGCAAACTCGTGAGCATGCTTTGCTTGCATTTACACTTGGTGTCAAGCAGATGATCTGTTGCTGCAACAAGATGGATGCTACAA
CCCCCAAGTACTCCAAGTCTAGATTCGAAGAAATTGTGAAGGAGGTTTCTTCTTATTTGAAGAAGGTTGGGTACAACCCCGACAAAATTGCT

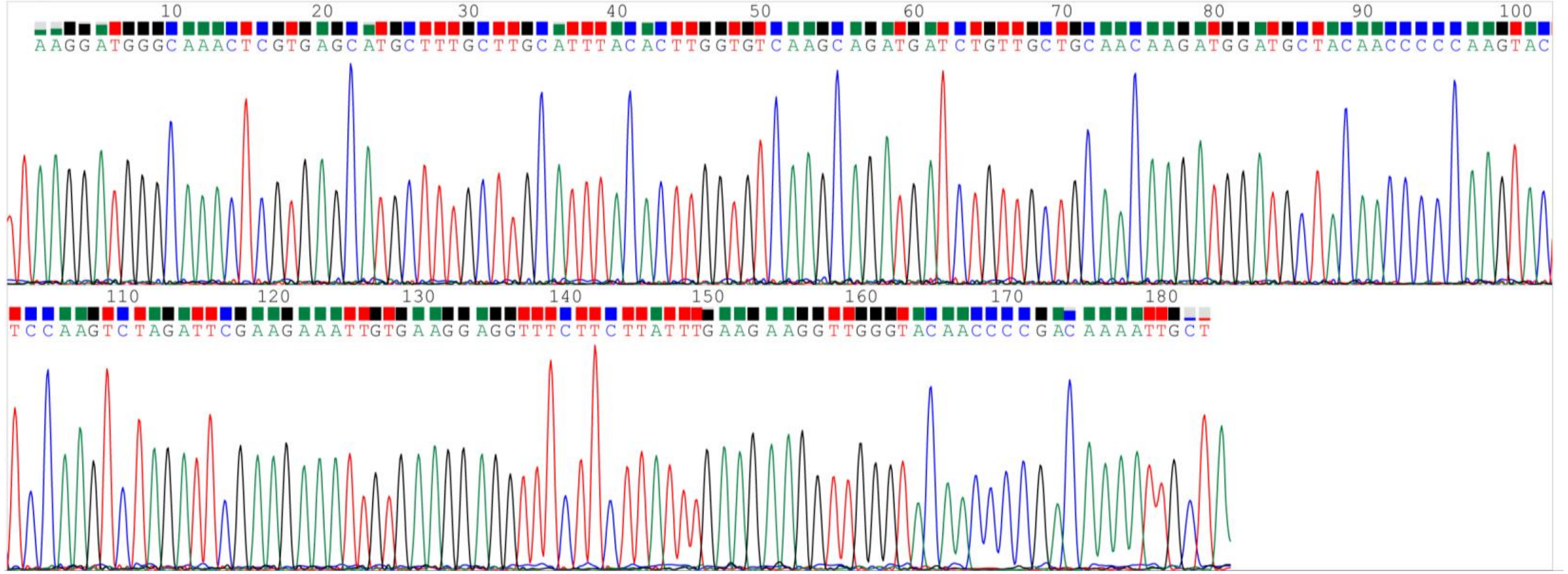

GAPDH

ACCTTCTTTGCACCTCCCTTCAAATGTGCAGCAGCCTTTTCCTTGTCAGTGAAGACACCAGTGGATTCAACAATATACTCTGCACCAGTGCT AGCCCATGGGATCTCCTCTGGGTTCCTGCAACCAAAGACAGC

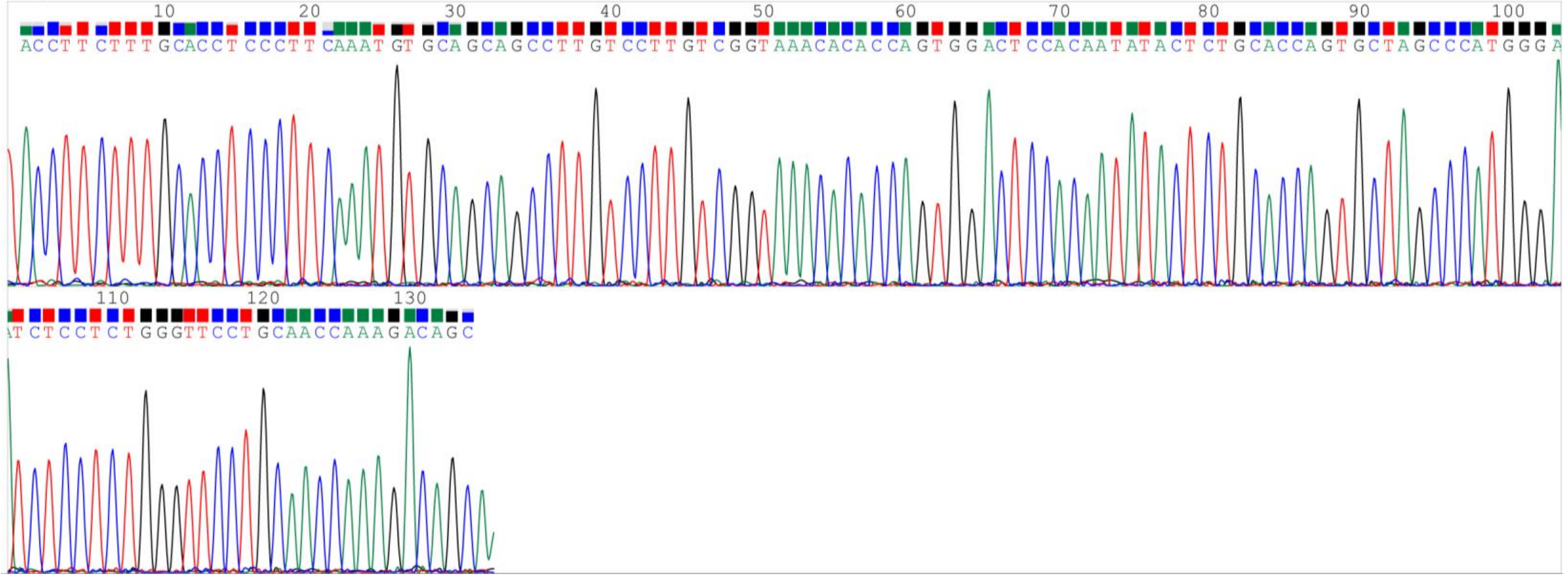

α-TUB

ACAACTTTGCTCGTGGACACTATACCATTGGAAAGGAGATTGTTGATCTTTGCCTGGATCGTATCAGGAAGCTTGCTGACAATTGCACTGGT
CTCCAGGGTTTCCTTGTTTTTAATGCTGTTGGAGGAGGCA

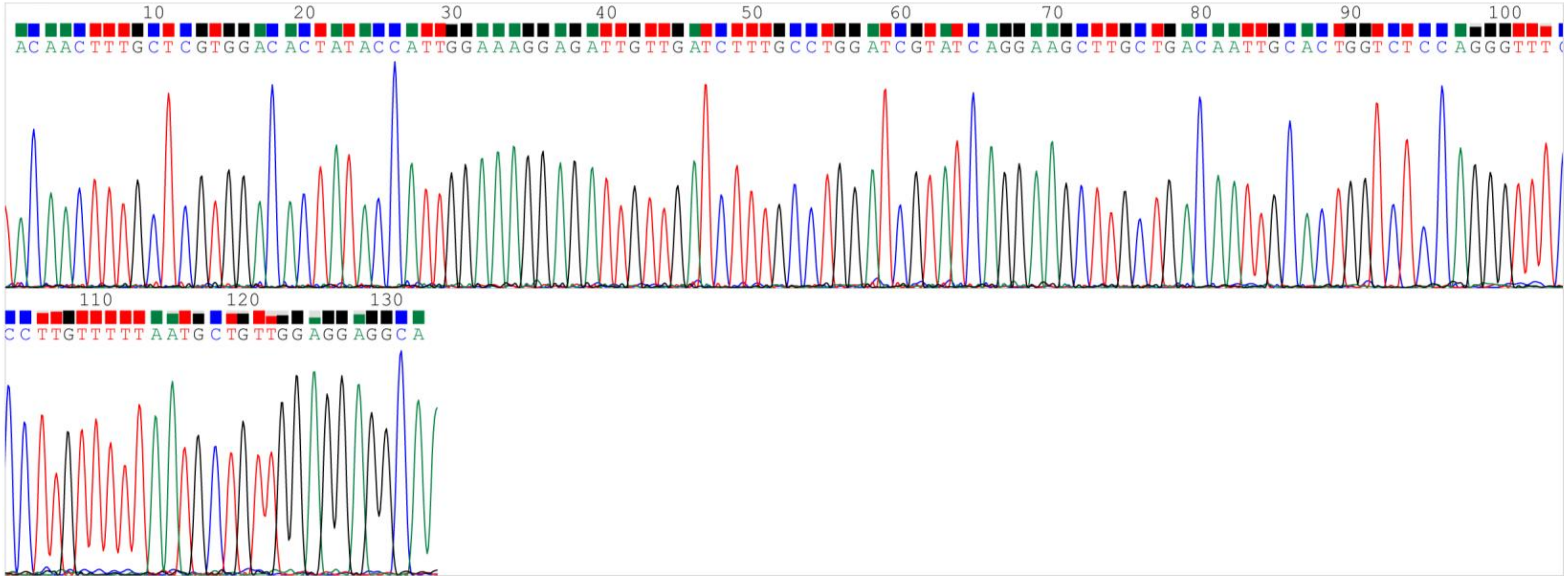

β-TUB

CAGGTACACTCAATGCACGGTATTGCTGGGAACCACGGGAGGTAAGAGGTGCAAATCCAACCATAAAGAAGTGCAACCTGGGGAAGGGAA
TGAGATTTACAGCCAACTTCCTGAGATCGGAGTTCAACTGACCAGGGAAACGCAAGCAGCATGTAACACCAGACATAGTGGCCGAAATCAA
GTGGTTTAGATCACCAAAGCTAGGTGTGGTAAGCTTCAAGGTGCG

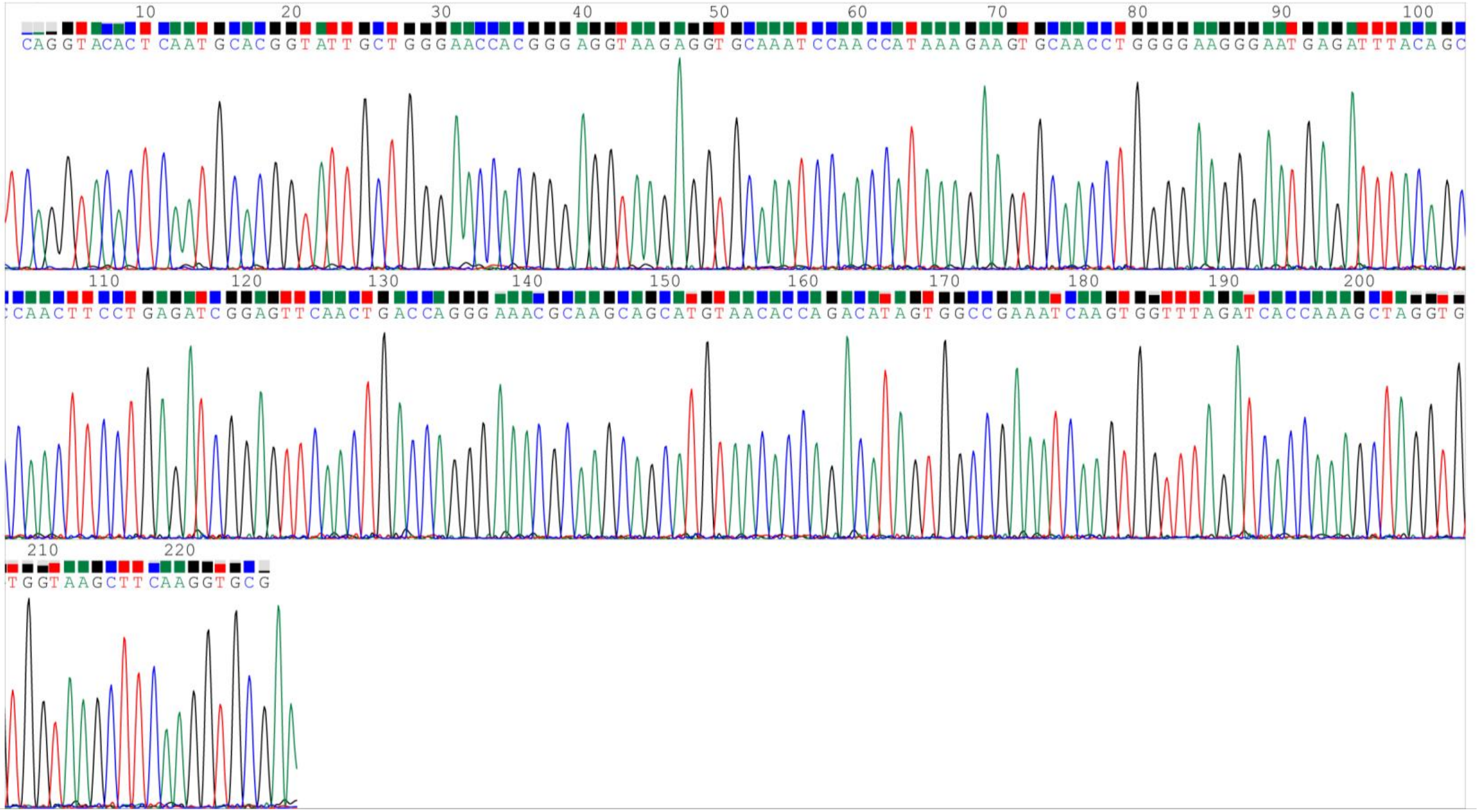

PTBP1

GGCTACGGTGTGATTGGAACTTGATGTTTAGATCTGTGTGAGCTGAATATGAGATGCGCAGATTGCAAGAACCAACATGTTCTGGAAGCAA
GTACCTTGGAATACTTCTGCCATCTAAAGCTTCCCTTGCTGAAAGAGCAGTCTCAGCATCAGTAAACTGGATTAGTGCCTGAAAACCTGCTG
CCTTCTCAAAAGTAGCAATCTTGTGCACAAATCCAAAAGCCGA

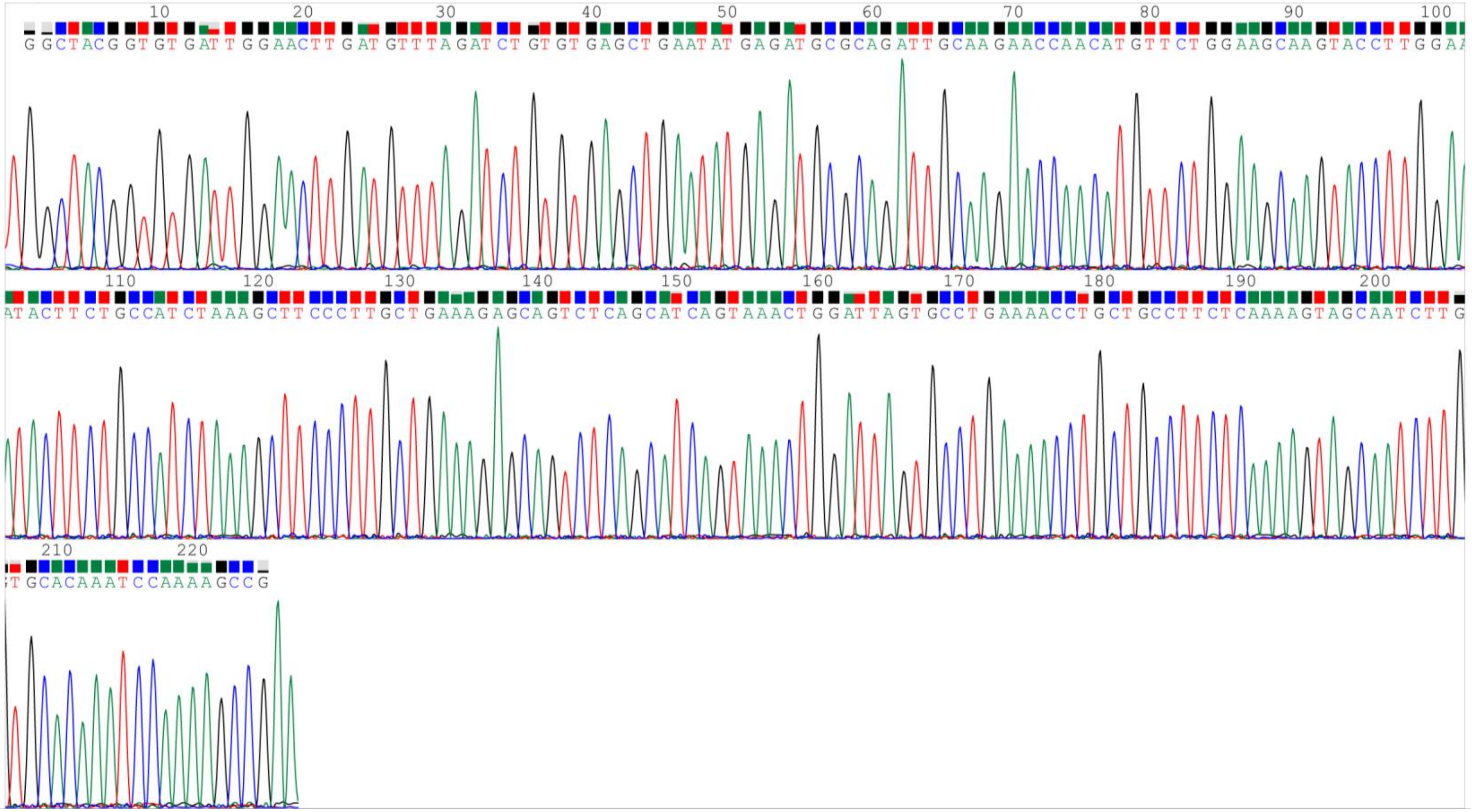

EXP1

AGCCCTCCTGCATGTTTAGTTCCGGGTGCTGGACTTGGTAGACTGGCTTTGGAAATTTCATGTCTTGGTTTTGCAAGCCAAGGAAATGAATT
TTCATACTATATGATGATCTGCTCGAGTTTTATTCTTAACCAAGCGGAAAGGGCTAATGAATGGACTATCCATCCTTGGATTCATAGCAATTG
CAATTCACTTTCTGACAGTGACCAGCTTCGT

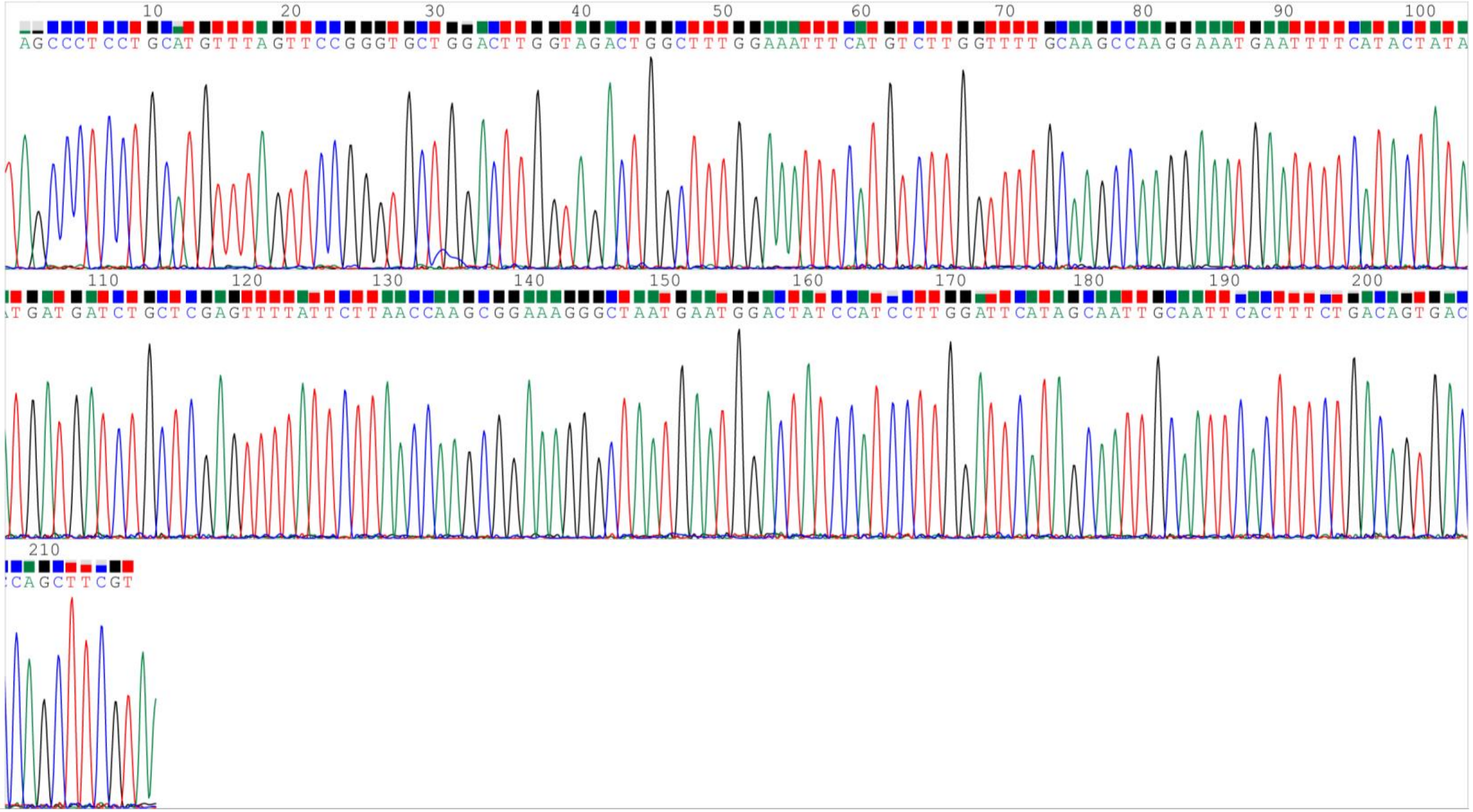

EXP2

ACACCCATATATTGCAGCGCTTTTCGCCTTCTCTTGCATTTTTGGCTTGATCCAGACAAGCTTGACAACAATCATAAGCGCTCTCTTTATGGT
GGGTAAGGCCCCAGCGAACAGCTGCCCCACCATAATCTGTATGAAGTTCAGTATGGCACTCGGGAGGAAGCTGTCTGCCGGCTAATATTT
CGCTGTCAAACTCTTCTTCACCCTCAGGCTTGTCATCATG

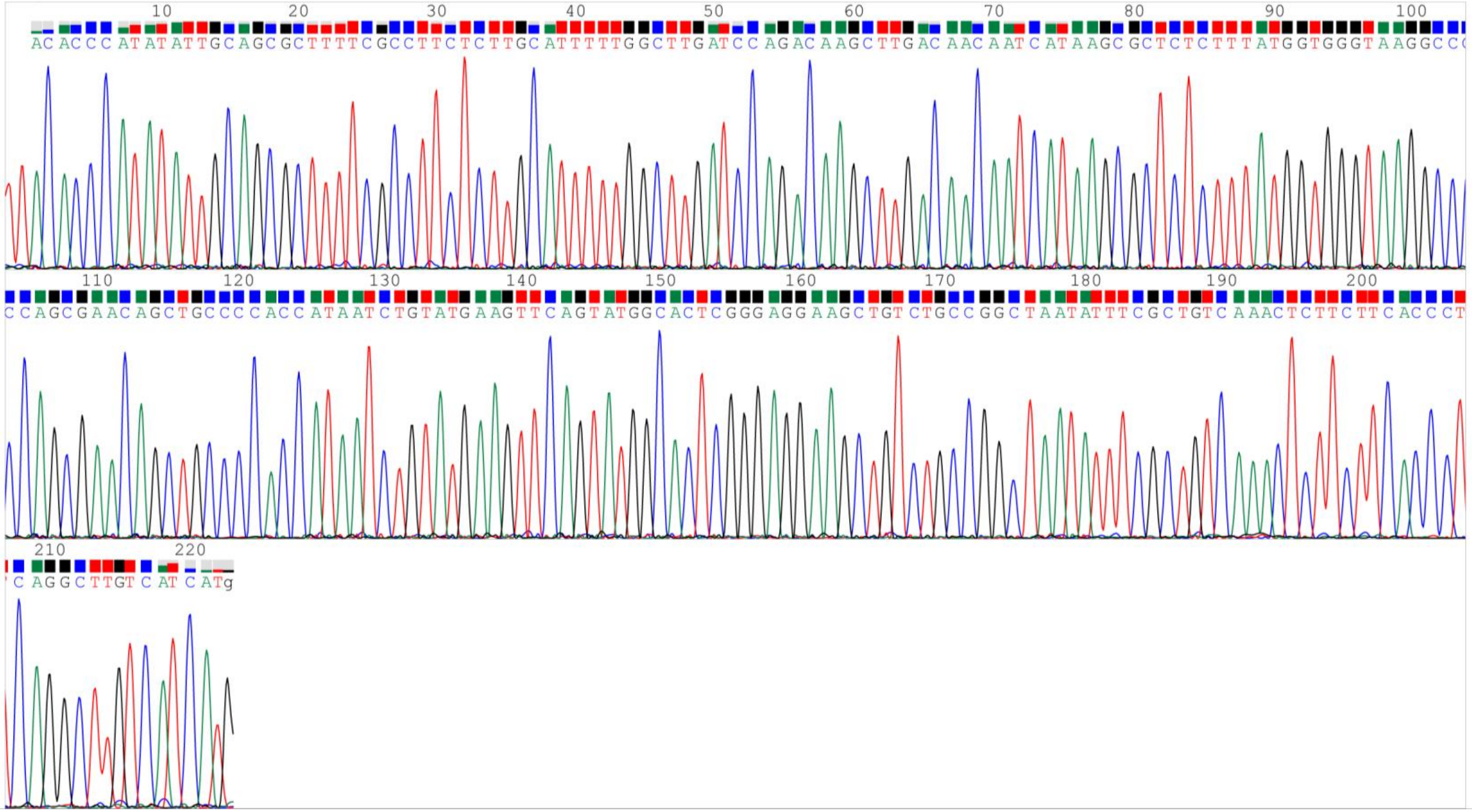

TIP41

AGAGTGATGCCAAGCTGTTGGTTTCTTCTCTTGCGTTTTTGGCTTAGAGTTGATGGTGTGCTTATGCGTTTAAGGGACACACGCATCCATTG

CATTTTTGGTGAGGGTAAAACACCAGTTATTCTGAGAGAATGTTGCTGGAGAGAGGC

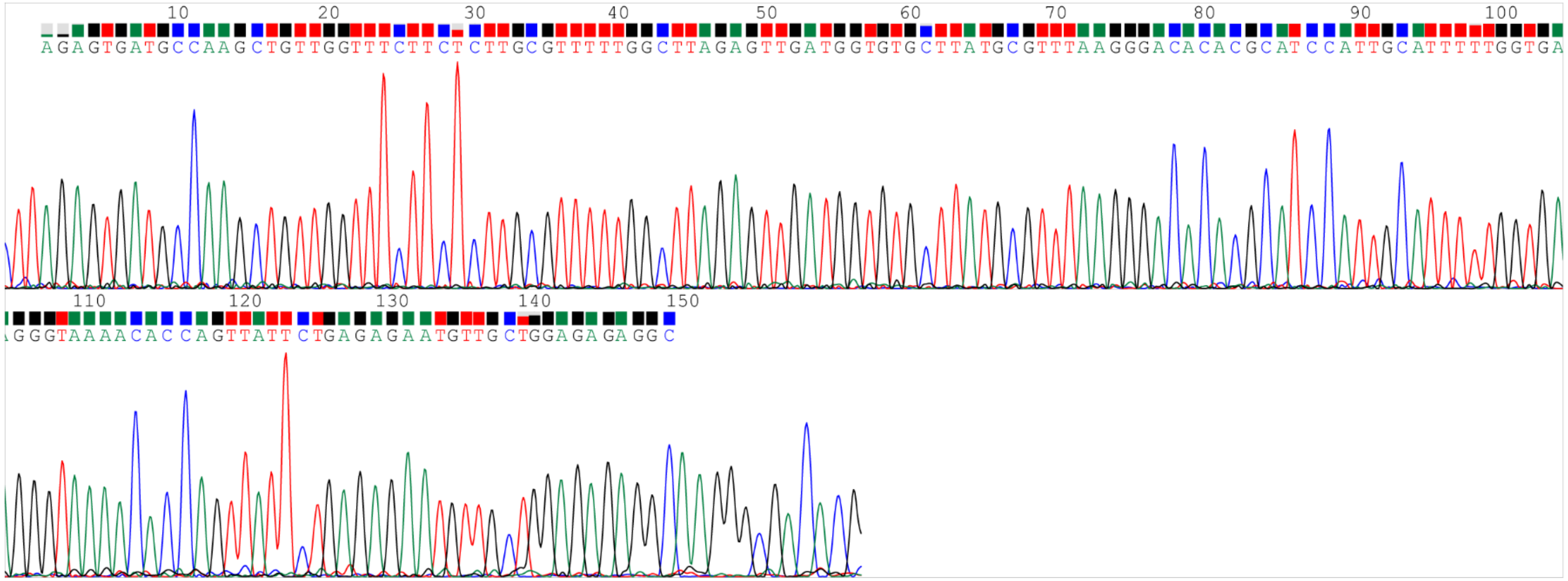

SAND

GGACCATTTCGATACGGATCCTCCAATCTTTTAGACGATGAAATGCATCAGGATTAGCAGTAAGCAACATCAAATAAGTATCAGCATCAAGA
TAATACACATAAGTATATAAAAATGCCATTGGATTGTATCTCGGAAGACAGATTGGAGAGAAAGATTCAGATGTCCTGAATGATTCAGATGA
CATCACAAAGTTGGCAAGCAAGAGCATATCATCGGGATGAAGAGACGCTT

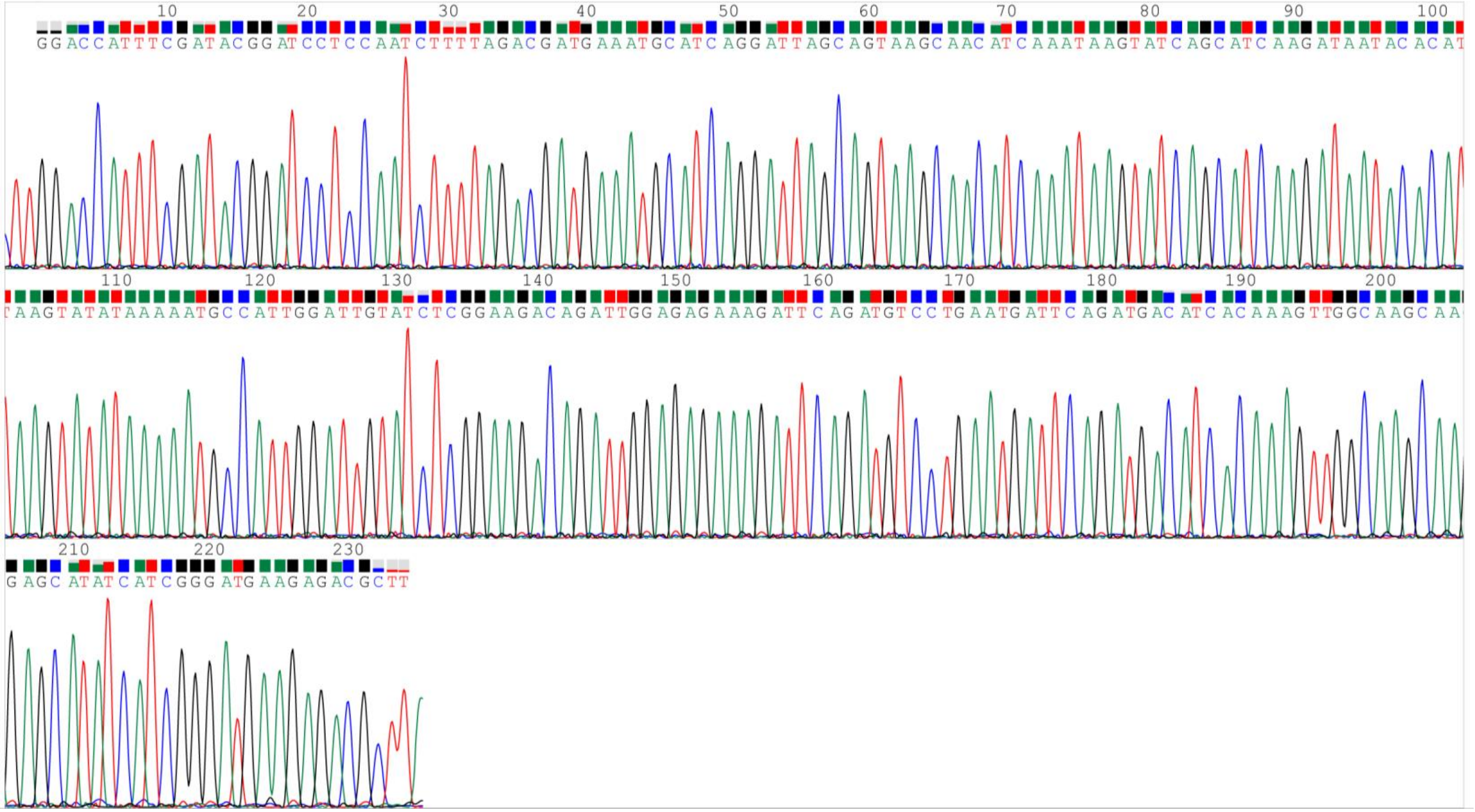

CYP2

GCCACTATCATTTGAGGAGACCAAAGCTTTAGCAGGGGCTGCCCTTGGAACAATATCATCAAAAGGGTTCCATATCACCTCAACTGAAATC
AATTTAGGGGGAGATTCTACTGGTCGATCATCCTTATCAGTTTCAACCTCGGAAAAGTTTAAGAGATTGTATAGTGAATCTCCAGTTACCTTT
CCGAAAATGGTATGTTTACGATCAAGCCAATCACAACG

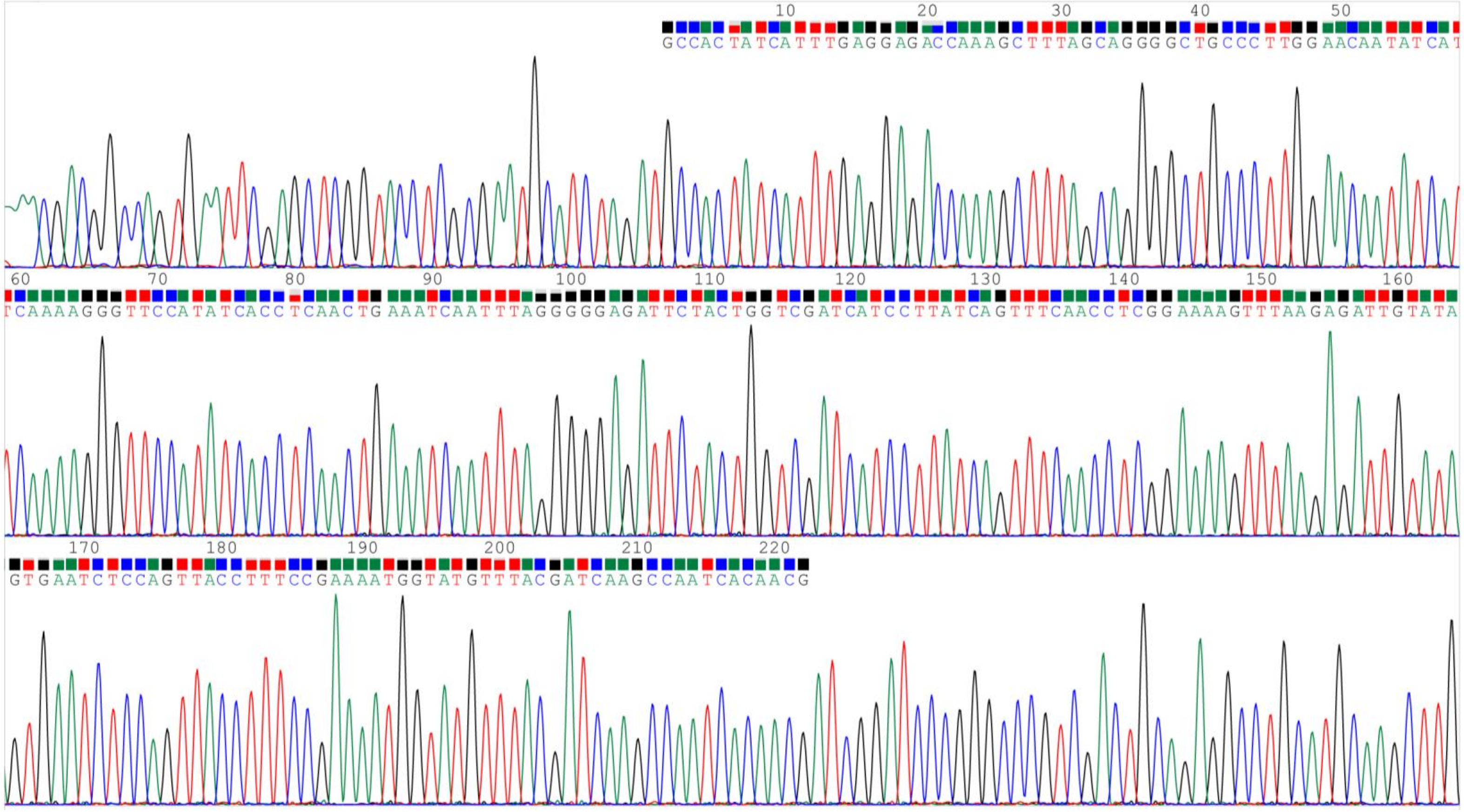

Supplement: Supplementary file 1 — Supplementary Information. [file 41598_2020_63917_MOESM1_ESM.pdf]
